# Four new species of *Pachymerium* centipedes from China (Geophilomorpha, Geophilidae)

**DOI:** 10.3897/zookeys.1281.182253

**Published:** 2026-06-08

**Authors:** Yifei Yu, Yuan Xiong, Chunxue You, Chao Jiang

**Affiliations:** 1 State Key Laboratory for Quality Ensurance and Sustainable Use of Dao-di Herbs, National Resource Center for Chinese Materia Medica, China Academy of Chinese Medical Sciences, Beijing, 100700, China Tianjin Agricultural University Tianjin China; 2 Tianjin Key Laboratory of Agricultural Animal Breeding and Healthy Husbandry, College of Animal Science and Veterinary Medicine, Tianjin Agricultural University, Tianjin 300392, China China Academy of Chinese Medical Sciences Beijing China

**Keywords:** Description, key, taxonomy

## Abstract

The genus *Pachymerium* is nearly cosmopolitan and exhibits considerable dispersal capacity among geophilomorph centipedes. However, its taxonomic study in China remains notably inadequate. Based on specimens resembling *Pachymerium* collected from 23 provincial-level administrative regions across China, this study confirms the occurrence of five species of *Pachymerium* in China, four of which are newly described: *P. dimorphum***sp. nov**., *P. proximiporum***sp. nov**., *P. multiseriale***sp. nov**. and *P. lingshouense***sp. nov**. A key to species of genus *Pachymerium* is provided.

## Introduction

The genus *Pachymerium* C.L. Koch, 1847 is nearly cosmopolitan and exhibits considerable dispersal capacity among geophilomorph centipedes (Popovici 2024). Based on a comprehensive review of ChiloBase and recent literature, 25 valid species are currently known (Bonato et al. 2011, 2016; Dyachkov et al. 2023; Foddai et al. 2000; Popovici 2024). Despite its wide distribution, records of *Pachymerium* in Asia remain sparse, with locations such as Azerbaijan, Armenia, the Caucasus, and Iraq documented in recent literature (Dyachkov et al. 2023; Dyachkov 2024, 2025; Evsyukov et al. 2025). As a result, the diversity and biogeography of this genus across the Asian continent are still poorly documented.

Prior to this study, only two species of *Pachymerium* had been recorded in China: the widely distributed *P. ferrugineum* (C.L. Koch, 1835), reported from Hualian and Shanxi, and the European *P. atticum* Verhoeff, 1901, also documented in Shanxi (Takakuwa and Takashima 1949; Wang 1956). The taxonomic status of the latter remains controversial as it may represent a valid species, many records outside the type locality are likely misidentified as *P. ferrugineum*. For example, Takakuwa and Takashima (1949) explicitly noted that specimens collected from Shanxi, identified as *P. atticum*, differ from the original description of the species.

In the present study, we examined 747 *Pachymerium*-like specimens collected from 93 sampling sites across 23 provincial-level administrative regions in China. A total of five species were identified, four of which are new to science. Although *P. multiseriale* sp. nov. and *P. lingshouense* sp. nov. differ from the type species of *Pachymerium* in the structure of the anterior part of the cephalic capsule, their assignment to the genus *Pachymerium* is considered provisional. This study aims to describe these new species, analyze the geographic distribution of *Pachymerium* in China, and provide an updated identification key to the currently recognized valid species of the genus.

## Materials and methods

Specimens were hand collected from Chinese mainland using tweezers between 2014 and 2025 and preserved in 75% ethanol. Specimens are deposited in National Resource Center for Chinese Materia Medica, China Academy of Chinese Medical Sciences, Beijing, China (**CMMI**).

Specimens were dissected and their cephalic plates, mandibles and maxillary complexes were mounted on temporary slides using 75% ethanol or lactic acid. Taxonomic characters were examined in lactic acid and photographed using a Leica M205 FCA stereomicroscope equipped with a Leica DMC 6200 camera. The photos were converted into hand-drawn illustrations with SKETCHBOOK 6.2.3. Localities were mapped with ArcMap 10.8. Morphological terminology for external anatomy follows Bonato et al. (2010).

## Taxonomy

### Order Geophilomorpha Pocock, 1895

**Family Geophilidae, Leach, 1815**

#### Genus Pachymerium

Taxon classificationAnimaliaGeophilomorphaGeophilidae

C.L. Koch, 1847

4CFC1B93-1DE5-5112-8F64-096BE30D7AA0

##### Type species.

*Geophilus ferrugineum* C.L. Koch, 1835 by monotypy.

##### Diagnosis.

See Popovici (2024).

#### Pachymerium ferrugineum

Taxon classificationAnimaliaGeophilomorphaGeophilidae

(C.L. Koch, 1835)

86E4CB6C-A8AC-52EC-8EB3-90AEEF1AC7CE

Figs 1A, 2A, 3

**Figure 1.**
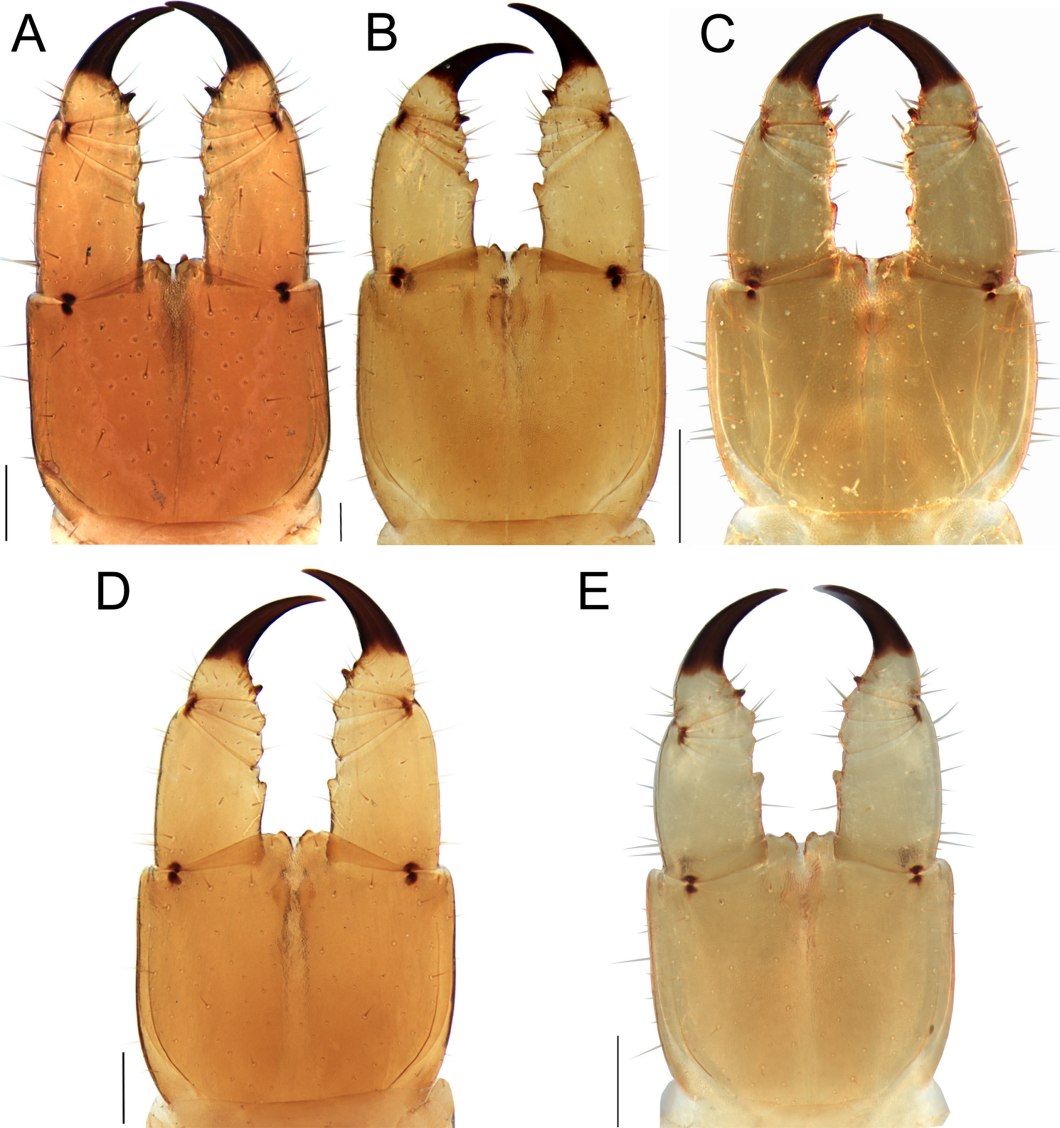
Forcipular segments. **A**. *Pachymerium ferrugineum* (C.L. Koch, 1835) (spm. CMMI 20240926002D); **B**. *P. dimorphum* sp. nov., holotype; **C**. *P. proximiporum* sp. nov., holotype; **D**. *P. lingshouense* sp. nov., holotype; **E**. *P. multiseriale* sp. nov., holotype. Scale bars: 250 μm.

**Figure 2.**
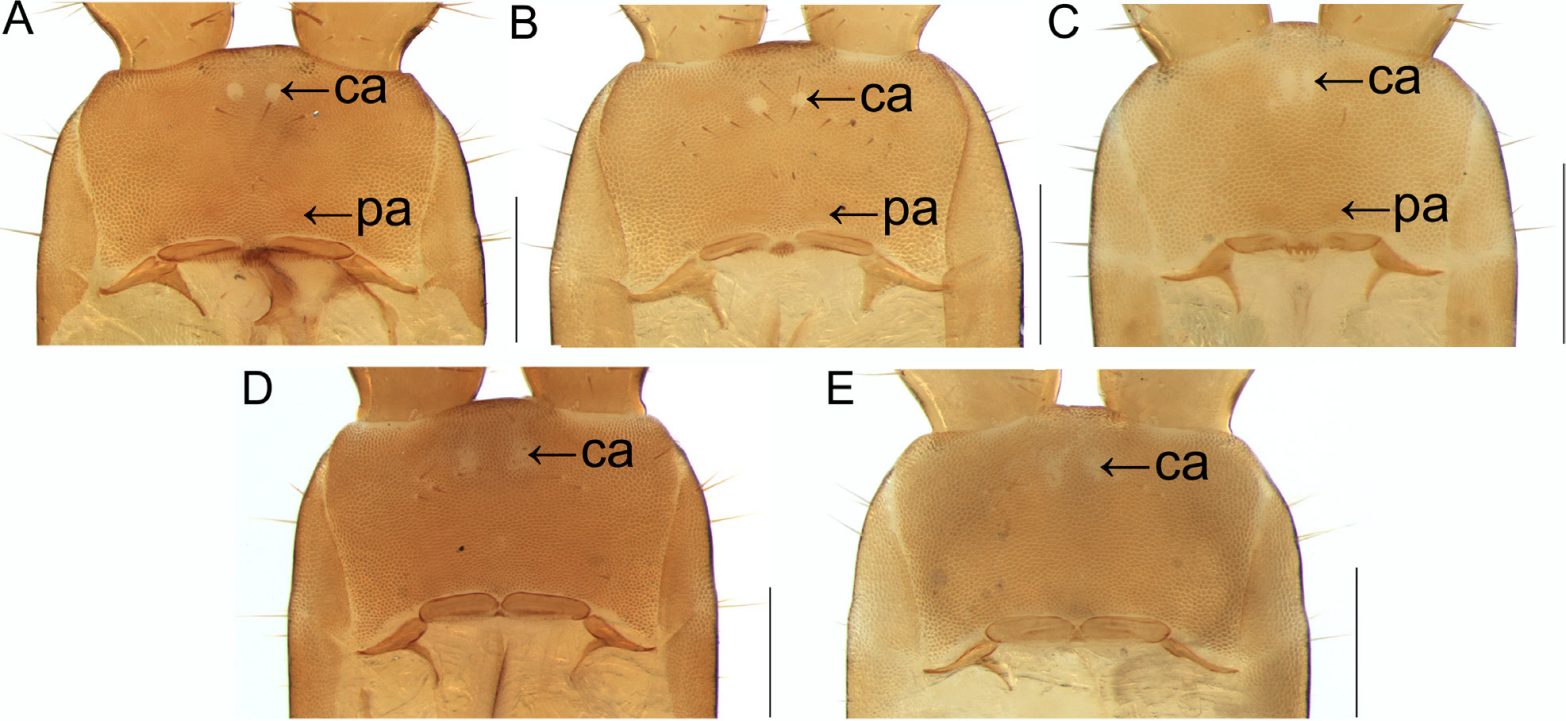
Clypeus and labrum. **A**. *P. ferrugineum* (C.L. Koch, 1835) (spm. CMMI 20240926002D); **B**. *P. dimorphum* sp. nov., holotype; **C**. *P. proximiporum* sp. nov., holotype; **D**. *P. lingshouense* sp. nov., holotype; **E**. *P. multiseriale* sp. nov., holotype. Scale bars: 250 μm. Abbreviations: ca – clypeal areas, pa – poorly defined, pigmented areas.

Geophilus ferrugineum C.L. Koch, 1835: 2.
Geophilus angustiventris Kessler, 1874: 44; Sseliwanoff, 1884: 78–80.
Geophilus caucasicus Attems, 1903: 256–257.
Geophilus felix Attems, 1947: 57, figs 24–26.
Geophilus kervillei Attems, 1908: 109; Attems 1929: 245.
Geophilus paradoxus Tömösváry, 1880: 619.
Arthronomalus puncticeps Lucas, 1849: 389.
Mecistocephalus punctilabium Newport, 1843: 179.
Pachymerium antipai Căpușe, 1968: 716; Popovici 2024: 590.
Pachymerium ferrugineum : Attems 1929: 245–246, fig. 205; Takakuwa and Takashima 1949: 58; Wang 1956: 158; Bonato et al. 2004: 25, figs 4, 12, 14; Dyachkov 2018: 253, 2022: 71; Popovici 2024: 590, 594, figs 1(A, B, D), 2; Bonato and Minelli 2014: 51.
Pachymerium ferrugineum helveticum Verhoeff, 1902: 559.
Pachymerium ferrugineum insulanum Verhoeff, 1902: 558.

##### Material examined.

670 specimens examined in this study are listed in Suppl. material 1.

**Figure 3.**
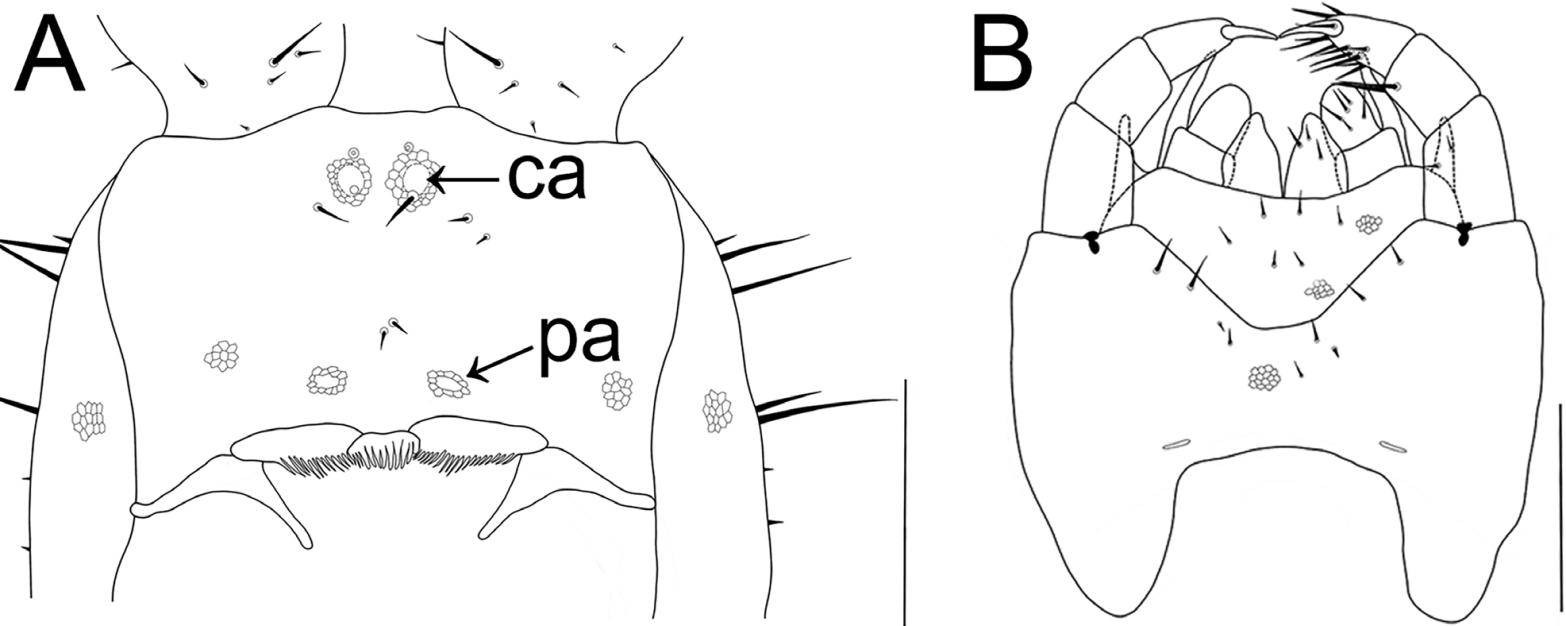
*Pachymerium ferrugineum* (C.L. Koch, 1835). **A**. Anterior part of cephalic capsule, ventral (maxillary complex removed); **B**. Maxillary complex, ventral (setae on right part omitted). Specimens: CMMI 20240926002D. Scale bars: 250 μm. Abbreviations: ca – clypeal areas, pa – poorly defined, pigmented areas.

##### Diagnosis.

Body length up to 60 mm; number of leg-bearing segments 39–63 (data on body length and number of leg-bearing segments compiled from Popovici 2024); clypeus with two clypeal areas and two poorly defined, pigmented areas close to posterior margin (Figs 2A, 3A). Labral mid-piece with 5–10 stout denticles; each side-piece entire, with *~* 15 bristles on lateral parts. Coxosternite of forcipular segment with anterior denticles; chitin lines incomplete, vanishing before reaching the condyle, *~* 0.6× as long as the coxosternite. Coxopleuron with *~* 30 coxal pores in adults, opening independently on ventral and dorsal sides of coxopleuron.

##### Remarks.

This species exhibits a broad distribution across the west Palearctic, often introduced throughout the world (Bonato et al. 2011). Most recent publications concerning this species primarily update its distribution records, and describe cases of teratology (Bonato et al. 2004; Dyachkov 2018, 2022; Popovici 2024). Records show that this species transcends conventional habitat boundaries, with confirmed records from coastal regions through xeric shrublands to frigid alpine zones. In this study, female specimens of *Pachymerium ferrugineum* significantly outnumbered males across all samples. Given that all collected specimens were confirmed as conspecific (i.e., *P. ferrugineum*), the observed sex ratio bias is unlikely to result from species misidentification. Temporal analysis revealed that the number of male individuals collected in March and July exceeded that in other months, suggesting a possible link to the centipede’s reproductive cycle. Furthermore, male specimens were encountered more frequently in China’s Oriental region than in the Palearctic region. In addition, specimens from the Oriental region exhibited smaller maximum body length compared to those from the Palearctic region. Although sampling was conducted exclusively in vegetation-rich habitats, significant differences exist between the Oriental and Palearctic regions in terms of climate, humidity, and temperature fluctuations. Therefore, we hypothesize that the overall female-biased sex ratio and the observed morphological variations in this study may be associated with sampling months (and thus the reproductive cycle) as well as environmental conditions.

##### Distribution.

China (Gansu, Guangdong, Henan, Hubei, Hunan, Jiangxi, Jilin, Liaoning, Shaanxi, Shanghai, Shanxi, Sichuan, Taiwan and Yunnan provinces, Guangxi Zhuang Autonomous Region, Ningxia Hui Autonomous Region, Xinjiang Uygur Autonomous Region, Xizang Autonomous Region); all of Europe; Afghanistan; Algeria; Armenia; Azerbaijan; Cape Verde; Caucasus; Chile; Iraq; Japan; Kyrgyzstan; Mongolia; Morocco; North America; Tajikistan; Uzbekistan; Turkmenistan (Attems 1929; Takakuwa and Takashima 1949; Bonato et al. 2004; Dyachkov 2018, 2022; Popovici 2024).

#### Pachymerium dimorphum sp. nov.

Taxon classificationAnimaliaGeophilomorphaGeophilidae

4E81113C-1E10-5518-B41F-60CEDB0AA6ED

https://zoobank.org/56017CEF-852F-4964-9372-5AEAC17DA544

Figs 1B, 2B, 4

##### Type material examined.

***Holotype*. China** • ♂ (CMMI 20240712002D), Xizang Autonomous Region, Lang County, Gagonggou (29.1420°N, 93.1330°E), 3090 m a.s.l., 12.vii.2024, leg. Chao Jiang & Qing Li. ***Paratypes*. China** • 1♀ (CMMI 20240712003D), same as holotype; 1♀ (CMMI 20240727001D), Xizang Autonomous Region, Nimu County, Nimu Township, G318 Highway (29.3480°N, 90.1730°E), 3700 m a.s.l., 27.vii.2024, leg. Chao Jiang; • 7♂♂6♀♀ (CMMI 20240716088D–091D, -093D–097D, -099D–102D), Qusong County, Qiuduojiang Township, S202 Highway (28.7860°N, 92.0970°E), 4460 m a.s.l., 16.vii.2024, leg. Chao Jiang & Qing Li.

**Figure 4.**
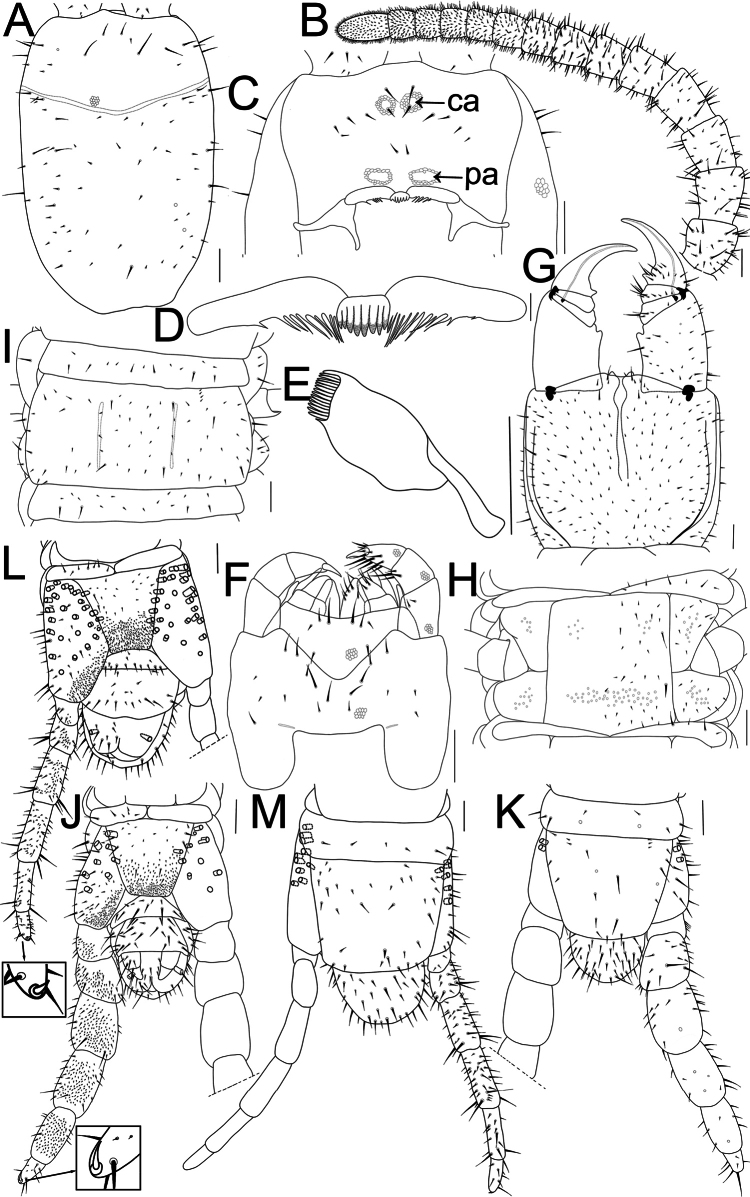
*Pachymerium dimorphum* sp. nov. **A**. Cephalic plate, dorsal (most part of antennae omitted); **B**. Right antenna, ventral; **C**. Anterior part of cephalic capsule, ventral (maxillary complex removed); **D**. Labrum; **E**. Mandible; **F**. Maxillary complex, ventral (setae on right part omitted); **G**. Forcipular segment, ventral (venom gland canal drawn with dashed lines; setae on right part omitted); **H**. Sternum of leg-bearing segment, ventral (setae on right part omitted); **I**. Tergite of leg-bearing segment, dorsal; **J**. Posterior end of body in adult male, ventral (setae on left coxopleuron and ultimate leg omitted); **K**. Ditto, dorsal; **L**. posterior end of body in adult female, ventral (setae on left coxopleuron and ultimate leg omitted); **M**. Ditto, dorsal. Areolation drawn in part in **A, C, F**. Specimens: holotype (**A–K**); Paratype ♀ (CMMI 20240712003D) (**L, M**). Scale bars: 250 μm (**A–C, E–M**); 20 μm (**D**). Abbreviations: ca – clypeal areas, pa – poorly defined, pigmented areas.

##### Other material.

**China** • 1♂2♀♀ (CMMI 20240717010D), Xizang Autonomous Region, Cuomei County, Near Cuomei County Hall, (28.4430°N, 91.4330°E), 4110 m a.s.l., 17.vii.2024, leg. Chao Jiang & Qing Li; • 3♂♂6♀♀ (CMMI 20230730004D–005D), Cuomei County, Naixi Township, G219 Highway (28.2562°N, 91.2160°E), 4340 m a.s.l., 30.vii.2023, leg. Chao Jiang.

##### Diagnosis.

Body length reaching at least 22 mm; number of leg-bearing segments 47–49(♂), 49–51(♀); clypeus with two distinct and internally non-areolate clypeal areas and two poorly defined, pigmented areas close to posterior margin (Fig. 2B); labral mid-piece with eight stout denticles, with *~* 10–13 bristles on lateral parts; coxosternite of forcipular segment with anterior denticles; chitin lines incomplete, vanishing before reaching the condyle, *~* 0.9× as long as the coxosternite; coxopleuron with 10–12(♂), 31–37(♀) coxal pores in adults, opening independently on ventral and dorsal sides of coxopleuron.

##### Description of holotype (adult male).

Body 22 mm long; with 49 leg-bearing segments; body tapering distally. Color (in ethanol 75%) pale orange; forcipules darker.

***Cephalic capsule*** (Fig. 4A, C, D) subrectangular; *~* 1.5× longer than wide, anterior margin convex; areolation uniform on the entire surface; with an indistinct transverse suture. Clypeus with two distinct and internally non-areolate clypeal areas and two poorly defined, pigmented areas close to posterior margin. Clypeal setae: one pair anterior, five pairs intermediate (1 pair within clypeal areas) and one pair posterior. Labral mid-piece wider than long, with eight stout denticles; each side-piece entire, with *~* 10–13 bristles on lateral parts.

***Antennae*** (Fig. 4B) almost uniform in width; *~* 1.3× as long as the length of the head. Basal articles only slightly more elongated (article II *~* 1.2× as long as wide); distal articles stouter (article XIII *~* 0.9× as long as wide); article XIV *~* 1.9× as long as wide. Setae gradually denser and shorter from the basal articles to the distal ones. Articles I–VII with distinctly three whorled long setae along with numerous short setae; remaining articles equipped with uniform setae.

***Mandible*** (Fig. 4E) with a single pectinate lamella with *~* 16 hyaline teeth.

***First maxillae*** (Fig. 4F). Coxosternite entire; uniformly areolate; with pair of evident lateral lappets; 4+4 setae close to the anterior margin. Coxal projection subtriangular; longer than wide; ventral side with 5+6 setae on distal half; dorsal surface with numerous small sensilla on distal half. Telopodite with two articles, telopodite as long as the coxal projection; distinctly articulated; one evident external lappet on each article; ventral side with 3+5 setae on distal half; dorsal surface with numerous small sensilla on distal half.

***Second maxillae*** (Fig. 4F). Coxosternite entire; uniformly areolate; anterior margin deeply concave; 10+8 setae scattered on the coxosternite; metameric pores opening close to posterior margin. Telopodite composed of three articles; gradually narrowing toward the tip; pretarsus of telopodite pointed, almost straight and gradually tapering on the telopodite.

***Forcipular segment*** (Fig. 4G) Tergite subtrapezoid; *~* 2.2× as wide as long. Coxosternite *~* 1.1× as wide as long on exposed part; anterior margin moderately projecting with respect to its condyles; anterior border with shallow medial concavity; with anterior denticles; chitin lines incomplete, vanishing before reaching the condyle, *~* 0.9× as long as the coxosternite. Trochanteroprefemur *~* 1.6× as long as wide. Forcipular articles from trochanteroprefemur to tarsungulum with 1, 0, 0, and 1 denticle, respectively; basal denticle of the tarsungulum is subtriangular. Tarsungulum *~* 2.4× as long as wide. Basal denticle of tarsungulum with distal margin distinctly convex and *~* 0.2× as long as the basal breadth of the tarsungulum. Calyx of venom gland short, *~* 1.2× as long as wide, situated in femur.

***Leg-bearing segments*** (Fig. 4H, I). Tergites of leg-bearing segments with two paramedian sulci, becoming faint or obsolete on posterior segments. Metasternites subrectangular; without a mid-longitudinal sulcus. Ventral pores begin on leg-bearing segment 1 as a transverse group (*~* 10 pores) close to posterior margin plus two paired pore-fields (*~* 5+6 pores) close to anterior margin; on segment 11 the transverse group replaced by two paired pore-fields (*~* 9+11 pores); absent from segments posterior to it and including segment 23. Leg pair 1 smaller than the others; pretarsus claw-like, reaching *~* 1/4 of the length of the tarsus.

***Ultimate leg-bearing segment*** (Fig. 4J, K). Pretergite *~* 4.3× as wide as long on exposed part. Metatergite *~* 1.2× as wide as long, subtrapezoid. Metasternite *~* 1.2× as wide as long; posterior margin *~* 0.4× as wide as anterior margin; with dense setae. Coxopleuron with 10–12 pores, opening independently, scattered on the ventral and dorsal sides of coxopleuron; setae on the coxopleuron distinctly denser close to the ventral posterior edge. Ultimate leg with dense setae on ventral and lateral sides. Ultimate pretarsus claw-like; *~* 0.3× as long as tarsus. Ultimate legs swollen with scattered setae; dense short setae on ventral sides.

***Postpedal segments*** (Fig. 4J). Intermediate sternite distinct and exposed; first genital sternite separated from pleurites by distinct sutures; gonopods biarticulate, with setae; penis conical; anal pores indistinct.

##### Differences in paratype (adult female).

Body 26–32 mm long; with 49–51 leg-bearing segments.

***Ultimate leg-bearing segment*** (Fig. 4L, M). Coxopleuron with 31–37 pores, opening independently, scattered on the ventral and dorsal sides of coxopleuron. Ultimate legs slender, with scattered setae; a few setae on ventral sides.

***Postpedal segments*** (Fig. 4L). Intermediate sternite distinct and exposed; first genital pleurosternite *~* 2.1× as wide as long, posterior margin slightly concave, uniformly with sparse setae; gonopodal lamina not distinctly bilobate, with sparse setae; anal pores present.

##### Differences in paratype (adult male).

Body 23–27 mm long; with 45–49 leg-bearing segments.

##### Etymology.

di-: a prefix meaning “two”, “double”, or “having two”. -morphus: a suffix meaning “form”, “shape” or “structure”, this term signifies that males and females of the species differ in the number of coxal pores. We suggest the Chinese common name as “异型地蜈蚣”.

##### Remarks.

This new species exhibits significant sexual dimorphism in the number of coxal pores, with females possessing a substantially greater count than males based on the available specimens. Given that coxal pore number may scale allometrically with body size and typically increases with age within a species, and considering that all observed females were larger than the males, it cannot be ruled out that the observed sexual dimorphism in pore count may be influenced by the younger age and smaller body size of the available male specimens. It resembles *Pachymerium ferrugineum* (C.L. Koch, 1835) in having the same number of clypeal areas (Attems 1929: fig. 205; Bonato et al. 2004: fig. 12) and two paired, poorly defined, pigmented areas close to posterior margin (Fig. 2B). However, it differs from *P. ferrugineum* in the number of clypeal setae, arrangement of the ventral pore-fields and morphology of the first maxillae. Specifically, *P. dimorphum* sp. nov. possesses seven pairs of clypeal setae (14 setae total), contrasting with the usual five pairs (10 setae total) in *P. ferrugineum*. The telopodite of the first maxillae is much longer than the coxal projection in *P. ferrugineum* (Popovici 2024: fig. 1D), whereas in the new species, it is only slightly longer, being nearly equal in length.

##### Distribution.

China (Xizang Autonomous Region).

#### Pachymerium proximiporum sp. nov.

Taxon classificationAnimaliaGeophilomorphaGeophilidae

D2A376F9-DBB2-528B-BB37-6FDDF5D7E8E4

https://zoobank.org/19C67675-F249-462B-BFFE-F1CE568D7431

Figs 1C, 2C, 5

##### Type material examined.

***Holotype*. China** • ♂ (CMMI 20230420003D), Chongqing, Wushan, Gaotang Street, Pinghu Road (31.0742°N, 109.8775°E), 200 m a.s.l., 20.iv.2023, leg. Chao Jiang. ***Paratypes*. China • Hunan Province** • 2♂♂ (CMMI 20250415026), Zhangjiajie, Yongding Dist., Huilong Park (29.1282°N, 110.5005°E), 220 m a.s.l., 15.iv.2025, leg. Yuan Xiong & Jing Zhong. **– Sichuan Province** • 1♀ (CMMI 20210415134D), Dujiangyan, Yukang Road (30.9662°N, 103.6129°E), 690 m a.s.l., 15.IV.2021, leg. Chao Jiang. • 1♀ (CMMI 20240416001D), Wanyuan, Taiping Town (32.0657°N, 108.0318°E), 610 m a.s.l., 16.iv.2024, leg. Chao Jiang; • 1♀ (CMMI 20240324009D), Suining, Mt. Hemingshan (36.5836°N, 105.5680°E), 300 m a.s.l., 24.iii.2024, leg. Yuan Xiong & Yifei Yu; • 1♂ (CMMI 20240322006D), Ziyang, Sanxian Park (30.1392°N, 104.6360°E), 360 m a.s.l., 22.iii.2024, leg. Yuan Xiong & Yifei Yu.

**Figure 5.**
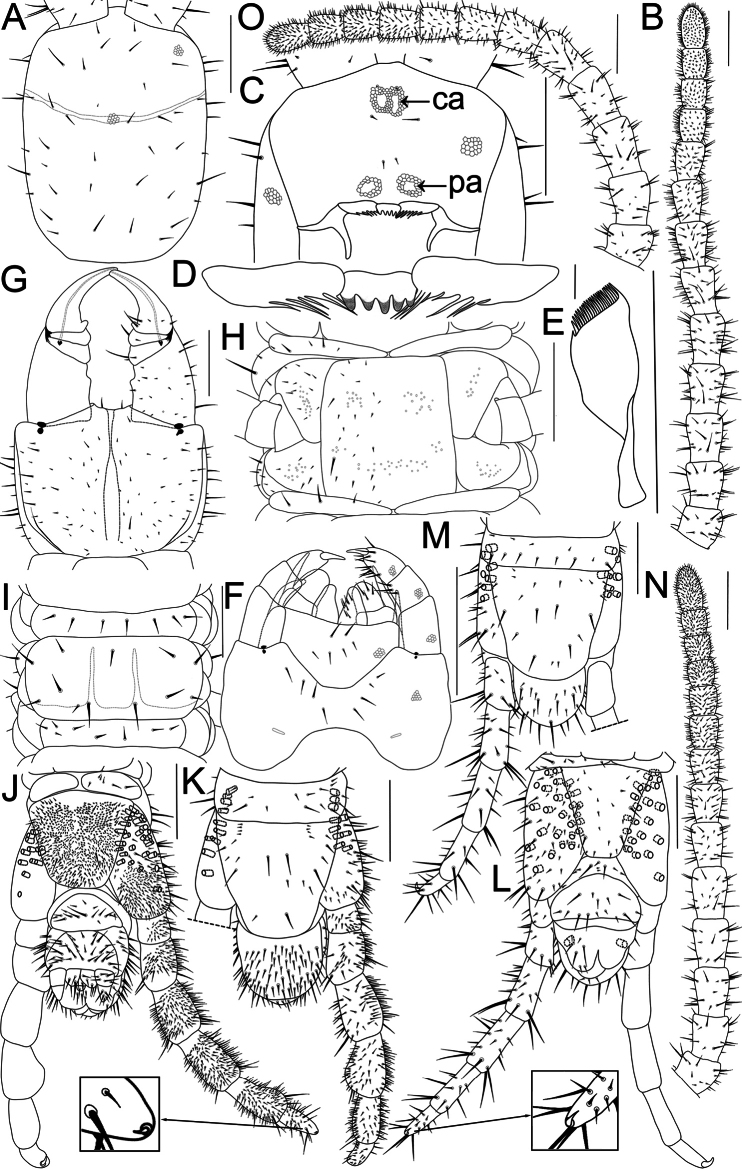
*Pachymerium proximiporum* sp. nov., holotype. **A**. Cephalic plate, dorsal (most part of antennae omitted); **B, N, O**. Antennae, ventral; **C**. Anterior part of cephalic capsule, ventral (maxillary complex removed); **D**. Labrum; **E**. Mandible; **F**. Maxillary complex, ventral (setae on right part omitted); **G**. Forcipular segment, ventral (venom gland canal drawn with dashed lines; setae on right part omitted); **H**. Sternum of leg-bearing segment, ventral (setae on left part omitted); **I**. Tergite of leg-bearing segment, dorsal; **J**. Posterior end of body in adult male, ventral (setae on right coxopleuron and ultimate leg omitted); **K**. Ditto, dorsal (setae on left coxopleuron and ultimate leg omitted); **L**. Posterior end of body in adult female, ventral (setae on left coxopleuron and ultimate leg omitted); **M**. Ditto, dorsal (setae on right coxopleuron and ultimate leg omitted). Specimens: holotype (**A–K, O**); Paratype ♀ (CMMI 20210419142) (**L–N**). Areolation drawn in part in **A, C, F**. Scale bars: 250 μm (**A–C, E–O**); 20 μm (**D**). Abbreviations: ca – clypeal areas, pa – poorly defined, pigmented areas.

##### Diagnosis.

Body length reaching at least 20 mm; number of leg-bearing segments 41–49; clypeus with two distinct and internally non-areolate clypeal areas and two poorly defined, pigmented areas close to posterior margin (Fig. 2C); without the lateral rows of setae that are present in most species of *Pachymerium*; labral mid-piece with five stout denticles, with *~* 6–9 bristles on lateral parts; coxosternite of forcipular segment without anterior denticle; chitin lines incomplete, vanishing before reaching the condyle, *~* 0.3× as long as the coxosternite; coxopleuron with *~* 22–37 coxal pores in adults, pores opening independently, clustered on proximal half of the ventral and dorsal sides of coxopleuron except the most posterior one which is displaced from all other pores.

##### Description of holotype (adult male).

Body 21 mm long; with 43 leg-bearing segments; body tapering distally. Color (in ethanol 75%) pale yellow; forcipules darker.

***Cephalic capsule*** (Fig. 5A, C, D) subrectangular; *~* 1.4× longer than wide; anterior margin convex; areolation uniform on the entire surface; with a distinct transverse suture. Clypeus with two distinct and internally non-areolate clypeal areas and two poorly defined, pigmented areas close to posterior margin. Clypeal setae: one pair anterior, one pair intermediate (clypeal areas without setae) and one pair posterior. Labral mid-piece wider than long, with five stout denticles; each side-piece entire, with *~* 6–9 bristles on lateral parts.

***Antennae*** (Fig. 5B, N, O) composed of 14 articles (occasionally exhibiting a developmental aberration with 12–13), almost uniform in width; *~* 3.9× as long as the length of the head. Basal articles only slightly more elongated (article III *~*1.3× as long as wide); distal articles stouter (article XII *~* 1.2× as long as wide); article XIII *~* 1.8× as long as wide. Setae gradually denser and shorter from the basal articles to the distal ones. Articles I–X with two whorls of long, distinct setae along with numerous short setae; remaining articles equipped with uniform short setae.

***Mandible*** (Fig. 5E) with a single pectinate lamella with *~* 24 hyaline teeth.

***First maxillae*** (Fig. 5F). Coxosternite entire; uniformly areolate; with a pair of lateral lappets; 3+3 setae close to the anterior margin. Coxal projection subtriangular; longer than wide; ventral side with 7+8 setae on distal half; dorsal surface with numerous small sensilla on distal half. Telopodite with two articles, telopodite longer than coxal projection; distinctly articulated; one external lappet on each article; ventral side with 4+4 setae on distal half; dorsal surface with numerous small sensilla on distal half.

***Second maxillae*** (Fig. 5F). Coxosternite entire; uniformly areolate; anterior margin deeply concave; 4+4 setae close to the anterior margin; metameric pores opening close to posterior margin. Telopodite composed of three articles; gradually narrowing toward the tip; pretarsus of telopodite pointed, almost straight and gradually tapering toward the tip.

***Forcipular segment*** (Fig. 5G). Tergite subtrapezoid; *~* 2.3× as wide as long. Coxosternite *~* 1.3× as wide as long on exposed part; anterior margin moderately projecting with respect to its condyles; anterior border with shallow medial concavity; without anterior denticles; chitin lines incomplete, vanishing before reaching the condyle, *~* 0.3× as long as the coxosternite. Trochanteroprefemur *~* 1.6× as long as wide. Forcipular articles from trochanteroprefemur to tarsungulum with 1, 0, 0, and 1 denticle, respectively; basal denticle of the tarsungulum is subtriangular. Tarsungulum *~* 2.6× as long as wide. Basal denticle of tarsungulum with distal margin distinctly convex and *~* 0.2× as long as the basal breadth of the tarsungulum. Calyx of venom gland short, *~* 1.4× as long as wide, situated in femur.

***Leg-bearing segments*** (Fig. 5H–I). Tergites of leg-bearing segments with two paramedian sulci, becoming faint or obsolete on posterior segments. Metasternites subrectangular; without a mid-longitudinal sulcus. Ventral pores begin on leg-bearing segment 1 as a transverse group (*~* 15 pores) close to posterior margin plus two paired pore-fields (*~* 7+8 pores) close to anterior margin; on segment 14 the transverse group replaced by two paired pore-fields (*~* 11+14 pores); absent from segment 16 and posterior. Leg pretarsus claw-like, reaching *~* 1/3 of the length of the tarsus.

***Ultimate leg-bearing segment*** (Fig. 5J, K). Pretergite *~* 3.4× as wide as long on exposed part. Metatergite *~* 1.1× as wide as long, subtrapezoid. Metasternite *~* 1.1× as wide as long; posterior margin *~* 0.5× as wide as anterior margin; with dense setae. Coxopleuron with 22–37 pores, opening independently, clustered on the proximal half of the ventral and dorsal sides of coxopleuron, except the most posterior pore, which is separate from all the other pores; setae on the coxopleuron distinctly denser close to the ventral posterior edge. Ultimate leg with dense setae on ventral and lateral sides. Ultimate pretarsus claw-like; ~ 0.2× as long as tarsus.

***Postpedal segments*** (Fig. 5J). Intermediate sternite distinct and exposed; first genital sternite separated from pleurites by distinct sutures; gonopods biarticulate, with setae; penis conical; anal pores indistinct.

##### Differences in paratype (adult female).

Body 20–27 mm long; with 41 leg-bearing segments.

***Ultimate leg-bearing segment*** (Fig. 5L, M). Coxopleuron with 28–34 pores, opening independently, scattered on the ventral and dorsal sides of coxopleuron. Ultimate legs slender, with scattered setae; a few setae on ventral sides.

***Postpedal segments*** (Fig. 5L). Intermediate sternite distinct and exposed; first genital pleurosternite *~* 2.1× as wide as long, posterior margin slightly concave, uniformly with sparse setae; gonopodal lamina not distinctly bilobate, with sparse setae; anal pores present.

##### Differences in paratype (adult male).

Body 24 mm long; with 41 leg-bearing segments.

##### Etymology.

Latin: *proximus =* near, *porum =* pore. The specific epithet refers to the coxal pores aggregated on proximal half of the coxopleuron ventral side. We suggest the Chinese common name as “近孔地蜈蚣”.

##### Remarks.

The new species resembles *P. ferrugineum* in the number of clypeal areas (Attems 1929: fig. 205; Bonato et al. 2004: fig. 12), but can be distinguished from the latter by a combination of morphological characteristics: the number of bristles on labral lateral parts, and the distribution of the coxal pores on the ventral side of the coxopleuron. The new species bears 6–9 bristles on the labral lateral parts, compared to 15 bristles in *P. ferrugineum* (Popovici 2024: fig. 1A). It also lacks the lateral rows of setae characteristic of most *Pachymerium* species. Regarding the ventral surface of the coxopleuron, the coxal pores in the new species are clustered mainly on the proximal half, with only the posteriormost pore situated more distally. In *P. ferrugineum*, the coxal pores are more broadly scattered across the ventral surface of the coxopleuron (Bonato et al. 2004: fig. 4). Furthermore, this species exhibits 12–14 antennal segments, and all examined individuals are adults. Although 14 antennal segments is the typical number for Geophilomorpha, the observed reduction is unlikely to result from postembryonic damage. As Minelli et al. (2000) pointed out, no reliable instance of antennal regeneration is known in Geophilomorpha, and they are inclined to think that no such regeneration actually occurs in this group. Therefore, specimens with fewer than 14 articles, especially when the terminal article retains the shape and sensilla characteristic of a typical article XIV, are more likely attributable to developmental defects (non-disjunction of potential antennomeres) rather than physical injury.

##### Distribution.

China (Hunan and Sichuan provinces; Chongqing).

#### Pachymerium multiseriale sp. nov.

Taxon classificationAnimaliaGeophilomorphaGeophilidae

AE0127CC-B1E8-5E19-86DF-E8E4E2253DF8

https://zoobank.org/2F976DA6-9CF6-4C4D-ABE7-1C17B7B04EF6

Figs 1E, 2E, 6

##### Type material examined.

***Holotype*. China** • ♂ (CMMI 20230920022D), Qinghai province, Xining, Near Xining High-Speed Rail Station (36.6158°N, 101.8241°E), 2220 m a.s.l., 20.ix.2023, leg. Tianyun Chen, Jiabo Fan & Yiying Zhao. ***Paratypes*. China** • 14♂♂4♀♀ (CMMI 20230920002D–006D, -008D, -013D–021D, -023D–024D), same as holotype.

**Figure 6.**
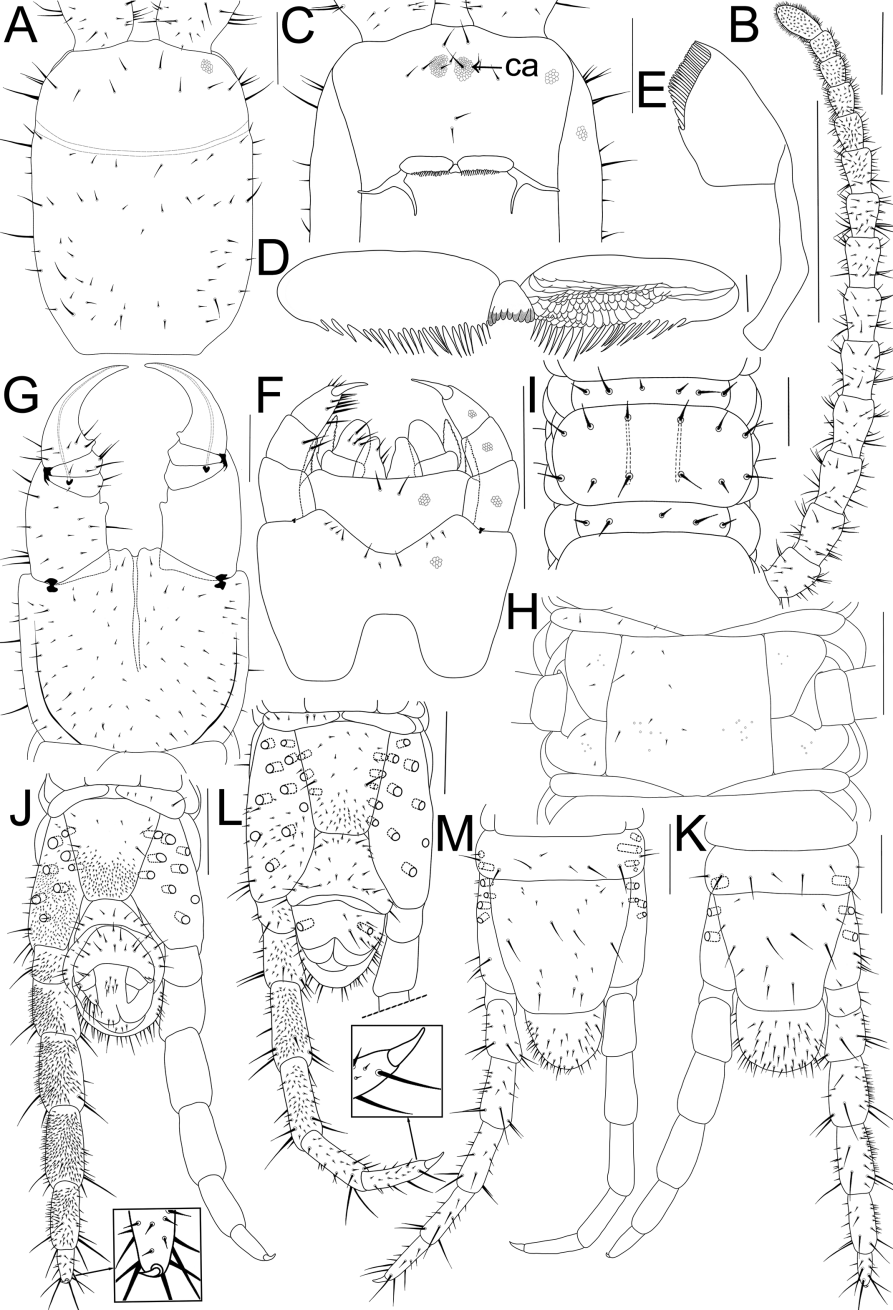
*Pachymerium multiseriale* sp. nov., holotype. **A**. Cephalic plate, dorsal (most part of antennae omitted); **B**. Right antenna, ventral; **C**. Anterior part of cephalic capsule, ventral (maxillary complex removed); **D**. Labrum; **E**. Mandible; **F**. Maxillary complex, ventral (setae on left part omitted); **G**. Forcipular segment, ventral (venom gland canal drawn with dashed lines; setae on left part omitted); **H**. Sternum of leg-bearing segment, ventral (setae on left part omitted); **I**. Tergite of leg-bearing segment, dorsal; **J**. Posterior end of body in adult male, ventral (setae on left coxopleuron and ultimate leg omitted); **K**. Ditto, dorsal; **L**. Posterior end of body in adult female, ventral (setae on left coxopleuron and ultimate leg omitted); **M**. Ditto, dorsal (setae on right coxopleuron and ultimate leg omitted). Areolation drawn in part in **A, C, F**. Specimens: holotype (**A–K**); Paratype ♀ (CMMI 20230920008D) (**L, M**). Scale bars: 250 μm (**A, C, E–M**); 500 μm (**B**); 20 μm (**D**). Abbreviation: ca – clypeal areas.

##### Other material.

**China • Henan Province** • 1♀ (CMMI 20240705045D), Yuzhou, near Yuzhou Forest Botanical Garden (34.1684°N, 113.4983°E), 120 m a.s.l., 05.vii.2024, leg. Jiabo Fan & Yizhan Shi; • 3♂♂1♀ (CMMI 20240702024D, -027D–029D), Linzhou, Feilong Gorge (36.1702°N, 113.6880°E), 700 m a.s.l., 02.vii.2024, leg. Jiabo Fan & Yizhan Shi. – **Heilongjiang Province** • 3♀♀ (CMMI 20240718014D, -032D, -054D), Shuangyashan, Heilongjiang Qingshan National Forest Park, Main Gate Parking Lot (46.5007°N, 131.1939°E), 260 m a.s.l., 18.vii.2024, leg. Jiabo Fan, Jing Zhong & Feiyu Huang; • 1♂4♀♀ (CMMI 20240701007D, -026D, -031D, -033D, -046D), Qiqihar, Longsha Park (47.3496°N, 123.9454°E), 150 m a.s.l., 1.vii.2024, leg. Yuan Xiong & Yifei Yu; • 1♂ (CMMI 20240628035D), Bin County, Xianglushan National Forest Park (45.5816°N, 127.5453°E), 340 m a.s.l., 28.vi.2024, leg. Yuan Xiong & Yifei Yu; – **Jilin Province** • 1♂ (CMMI 20240926002D), Tonghua County, Jindou Korean-Manchu Ethnic Township, Luojiagou (41.7407°N, 125.6516°E), 800 m a.s.l., 26.ix.2024, leg. Chao Jiang; • 17♂♂16♀♀ (CMMI 20240924016D–018D), Tonghua, Dongchang Dist., Mt. Yuhuangshan (41.7316°N, 125.9380°E), 830 m a.s.l., 24.ix.2024, leg. Chao Jiang; • 5♂♂4♀♀ (CMMI 20250924002, -045, -047 -049–050, -053, -056, -058, -065), Yanbian Korean Autonomous Prefecture, Hunchun, Henandong Street (42.8657°N, 130.3756°E), 70 m a.s.l., 24.ix.2025, leg. Chao Jiang; • 1♂3♀♀ (CMMI 20250923091, -093, -099, -104), Yanbian Korean Autonomous Prefecture, Yanji, Maoershan National Forest Park (42.8427°N, 129.4603°E), 460 m a.s.l., 23.ix.2025, leg. Chao Jiang; • 1♂1♀ (CMMI 20240703034D, -048D), Songyuan, Baerda Park (45.1627°N, 124.8679°E), 120 m a.s.l., 03.vii.2024, leg. Yuan Xiong & Yifei Yu. **– Shaanxi Province** • 1♂ (CMMI 20190907031), Baoji, Mei County, Honghegu Forest Park (34.0869°N, 107.7461°E), 1010 m a.s.l., 07.ix.2019, leg. Chao Jiang. – **Shanxi Province** • 4♂♂ (CMMI 20231030003D–006D), Taiyuan, Longcheng Forest Park (37.9285°N, 112.7630°E), 1600 m a.s.l., 30.X.2023, leg. Tianyun Chen, Yuan Xiong & Jiabo Fan.

##### Diagnosis.

Body length reaching at least 12 mm; number of leg-bearing segments 47–49; clypeus with two indistinct and internally areolate clypeal areas; lacking poorly defined, pigmented, weakly areolate areas close to posterior margin; labral mid-piece with stout denticles, with *~* 20–22 bristles on lateral parts and seven or eight rows of dentate projections; coxosternite of forcipular segment with anterior denticles; chitin lines incomplete, vanishing before reaching the condyle, *~* 0.7× as long as the coxosternite; without pore-fields near the anterior margin of the metasternites; coxopleuron with 11–21 coxal pores in adults, opening independently on ventral and dorsal sides of coxopleuron.

##### Description of holotype (adult male).

Body 32 mm long; with 49 leg-bearing segments; body tapering distally. Color (in ethanol 75%) yellow or ochre yellow; forcipules darker.

***Cephalic capsule*** (Fig. 6A, C, D) subrectangular; *~* 1.4× longer than wide; anterior margin convex; areolation uniform on the entire surface; with an indistinct transverse suture. Clypeal areas indistinct and internally areolate; without poorly defined, pigmented, weakly areolate areas (Fig. 2E). Clypeal setae: one pair anterior, four or five pairs intermediate, one pair posterior. Labral mid-piece as wide as long, with nine stout denticles; with *~* 20–22 bristles and seven or eight rows of dentate projections on lateral parts.

***Antennae*** (Fig. 6B) almost uniform in width; *~* 3.4× as long as the length of the head. Basal articles only slightly more elongated (article II *~* 1.3× as long as wide); distal articles stouter (article XIII *~* 1.2× as long as wide); article XIV *~* 2.0× as long as wide. Setae gradually denser and shorter from the basal articles to the distal ones. Articles I–XII with distinctly two whorled long setae along with numerous short setae; remaining articles equipped with uniform setae.

***Mandible*** (Fig. 6E) with a single pectinate lamella with *~* 28 hyaline teeth.

***First maxillae*** (Fig. 6F). Coxosternite entire; uniformly areolate; with pair a of lateral lappets; 1+1 setae close to the anterior margin. Coxal projection subtriangular; as long as wide; ventral side with 3+3 setae on distal half; dorsal surface with numerous small sensilla on distal half. Telopodite with two articles, telopodite longer than coxal projection; distinctly articulated; one external lappet on each article; ventral side with 5+6 setae on distal half; dorsal surface with numerous small sensilla on distal half.

***Second maxillae*** (Fig. 6F). Coxosternite entire; uniformly areolate; anterior margin deeply concave; 5+5 setae on the middle part; metameric pore indistinct. Telopodite composed of three articles; gradually narrowing toward the tip; pretarsus of telopodite pointed, almost straight and gradually tapering on the telopodite.

***Forcipular segment*** (Fig. 6G). Tergite subtrapezoid; *~* 2.6× as wide as long. Coxosternite *~* 1.1× as wide as long on exposed part; anterior margin moderately projecting with respect to its condyles; anterior border with shallow medial concavity; with anterior denticles; chitin lines incomplete, vanishing before reaching the condyle, *~* 0.7× as long as the coxosternite. Trochanteroprefemur *~* 1.5× as long as wide. Forcipular articles from trochanteroprefemur to tarsungulum with 1, 0, 0, and 1 denticle, respectively; basal denticle of the tarsungulum is subtriangular. Tarsungulum *~* 2.8× as long as wide. Basal denticle of tarsungulum with distal margin distinctly convex and *~* 0.3× as long as the basal breadth of the tarsungulum. Calyx of venom gland short, *~* 1.1× as long as wide, situated in femur.

***Leg-bearing segments*** (Fig. 6H, I). Tergites of leg-bearing segments with two paramedian sulci, becoming faint or obsolete on posterior segments. Metasternites subrectangular; without a mid-longitudinal sulcus. Ventral pores begin on leg-bearing segment 1 as two pairs (*~* 8 pores) close to posterior margin; absent from segment 4 and posterior. Legs 1 smaller than the others; pretarsus claw-like, reaching *~* 1/3 of the length of the tarsus.

***Ultimate leg-bearing segment*** (Fig. 6J, K). Pretergite *~* 3.7× as wide as long on exposed part. Metatergite *~* 1.2× as wide as long, subtrapezoid. Metasternite *~* 1.0× as wide as long; posterior margin *~* 0.4× as wide as anterior margin; with dense setae. Coxopleuron with 11 or 12 pores, opening independently, scattered on the ventral and dorsal sides of coxopleuron; setae on the coxopleuron distinctly denser close to the ventral posterior edge. Ultimate leg with dense setae on ventral and lateral sides. Ultimate pretarsus claw-like; *~* 0.4× as long as tarsus. Ultimate legs swollen, with scattered setae; dense short setae on ventral sides.

***Postpedal segments*** (Fig. 6J). Intermediate sternite distinct and exposed; first genital sternite separated from pleurites by distinct sutures; gonopods biarticulate, with setae; penis conical; anal pores indistinct.

##### Differences in paratype (adult female).

Body 27–30 mm long; with 49 leg-bearing segments.

***Ultimate leg-bearing segment*** (Fig. 6L, M). Coxopleuron with 19–21 pores, opening independently, scattered on the ventral and dorsal sides of coxopleuron. Ultimate legs slender, with scattered setae; a few setae on ventral sides.

***Postpedal segments*** (Fig. 6L). Intermediate sternite distinct and exposed; first genital pleurosternite *~* 1.8× as wide as long, posterior margin slightly concave, uniformly with sparse setae; gonopods lamina distinctly bilobate, with sparse setae; anal pores present.

##### Differences in paratype (adult male).

Body 24–33 mm long; with 47–49 leg-bearing segments.

##### Etymology.

Latin: *multi* = many, *serialis* = series. The specific epithet refers to the lateral parts of the labrum bearing several layers of dentate projections. We suggest the Chinese common name as “层齿地蜈蚣”.

##### Remarks.

The temporary assignment of this species to the genus *Pachymerium* is proposed. In overall morphology, it closely resembles the type species of the genus, *Pachymerium ferrugineum*, but differs in several distinctive characters: it possesses a narrow, subtriangular labrum, which is characterized by seven or eight rows of dentate projections on its lateral parts, a feature not observed in any known species of *Pachymerium*. Additionally, it lacks pore-fields near the anterior margin of the metasternites and lacks poorly defined, pigmented, weakly areolate areas on the anterior cephalic capsule, both of which distinguish it from *P. ferrugineum*. Females have more coxal pores than males, a difference likely scaling with body size, since most females examined are larger.

##### Distribution.

China (Qinghai, Henan, Heilongjiang, Jilin, Shaanxi, and Shanxi provinces).

#### Pachymerium lingshouense sp. nov.

Taxon classificationAnimaliaGeophilomorphaGeophilidae

12490B85-9FDE-5B32-8283-A95CD96E6F29

https://zoobank.org/3240A869-1AEF-4923-8F3F-B1297744A19A

Figs 1D, 2D, 7

##### Type material examined.

***Holotype*. China** • ♂ (CMMI 20230920007D), Hebei Province, Lingshou, Wuyuezhai National Forest Park, 20.ix.2023, leg. Huiqin Ma. ***Paratypes*. China** • 4♂♂5♀♀ (CMMI 20140921002D, 20140927001D–005D, 20230920009D–011D), same locality as holotype, 20.ix.2023, 21–27.ix.2014, leg. Huiqin Ma.

**Figure 7.**
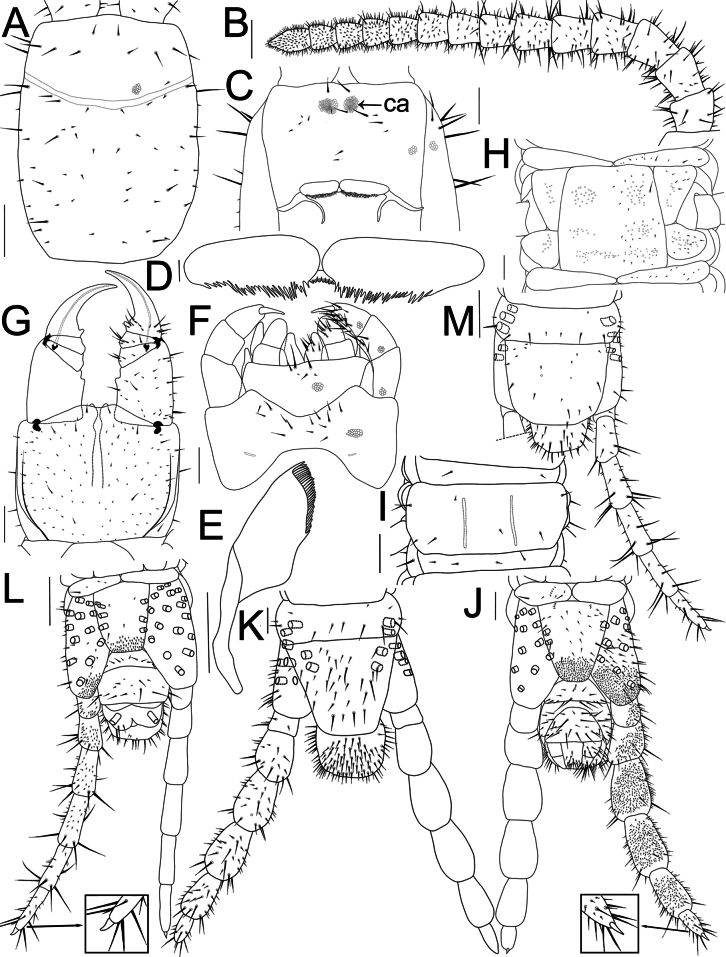
*Pachymerium lingshouense* sp. nov. **A**. Cephalic plate, dorsal (most part of antennae omitted); **B**. Left antenna, dorsal; **C**. Anterior part of cephalic capsule, ventral (maxillary complex removed); **D**. Labrum; **E**. Mandible; **F**. Maxillary complex, ventral (setae on right part omitted); **G**. Forcipular segment, ventral (venom gland canal drawn with dashed lines; setae on right part omitted); **H**. Sternum of leg-bearing segment, ventral (setae on right part omitted); **I**. Tergite of leg-bearing segment, dorsal; **J**. Posterior end of body in adult male, ventral (setae on right coxopleuron and ultimate leg omitted); **K**. Ditto, dorsal; **L**. Posterior end of body in adult female, ventral (setae on left coxopleuron and ultimate leg omitted); **M**. Ditto, dorsal. Areolation drawn in part in **A, C, F**. Specimens: holotype (**A–K**); Paratype ♀ (CMMI 20140921002D) (**L, M**). Scale bars: 250 μm (**A–C, E–M**); 20 μm (**D**). Abbreviation: ca – clypeal areas.

##### Diagnosis.

Body length reaching at least 28 mm; number of leg-bearing segments 41–49; clypeus with two indistinct and internally areolate clypeal areas; lacking poorly defined, pigmented, weakly areolate areas close to posterior margin (Fig. 2D); labral mid-piece with seven stout denticles, each side-piece entire, with *~* 25–27 bristles on lateral parts; coxosternite of forcipular segment with anterior denticles; chitin lines incomplete, vanishing before reaching the condyle, *~* 0.7× as long as the coxosternite; coxopleuron with 18–23(♂), 21–25(♀) coxal pores in adults, opening independently on ventral and dorsal sides of coxopleuron.

##### Description of holotype (adult male).

Body 37 mm long; with 49 leg-bearing segments; body tapering distally. Color (in ethanol 75%) pale orange; forcipules darker.

***Cephalic capsule*** (Fig. 7A, C, D) subrectangular; *~* 1.5× longer than wide; anterior margin convex; areolation uniform on the entire surface; with an indistinct transverse suture. Clypeal areas indistinct and internally areolate; without poorly defined, pigmented, weakly areolate areas. Clypeal setae: one pair anterior, 4–5 pairs intermediate, one pair posterior. Labral mid-piece wider than long, with seven stout denticles; with *~* 25–27 bristles on lateral parts.

***Antennae*** (Fig. 7B) almost uniform in width; *~* 3.0× as long as the length of the head. Basal articles only slightly more elongated (article II *~* 1.1× as long as wide); distal articles stouter (article XIII *~* 1.0× as long as wide); article XIV *~* 2.0× as long as wide. Setae gradually denser and shorter from the basal articles to the distal ones. Articles I–XIII with two whorls of long, distinct setae along with numerous short setae; remaining articles equipped with uniform short setae.

***Mandible*** (Fig. 7E) with a single pectinate lamella with *~* 28 hyaline teeth.

***First maxillae*** (Fig. 7F). Coxosternite entire; uniformly areolate; with pair a of lateral lappets; 3+3 setae close to the anterior margin. Coxal projection subtriangular; longer than wide; ventral side with 6+7 setae on distal half; dorsal surface with numerous small sensilla on distal half. Telopodite with two articles, telopodite longer than coxal projection; distinctly articulated; one external lappet on each article; ventral side with 11+7 setae on distal half; dorsal surface with numerous small sensilla on distal half.

***Second maxillae*** (Fig. 7F). Coxosternite entire; uniformly areolate; anterior margin deeply concave; 6+8 setae on the middle part; metameric pores opening close to posterior margin. Telopodite composed of three articles; gradually narrowing towards the tip; pretarsus of telopodite pointed, almost straight and gradually tapering towards the tip.

***Forcipular segment*** (Fig. 7G). Tergite subtrapezoid; *~* 2.1× as wide as long. Coxosternite *~* 1.2× as wide as long on exposed part; anterior margin moderately projecting with respect to its condyles; anterior border with shallow medial concavity; with anterior denticles; chitin lines incomplete, vanishing before reaching the condyle, *~* 0.7× as long as the coxosternite. Trochanteroprefemur *~* 1.9× as long as wide. Forcipular articles from trochanteroprefemur to tarsungulum with 1, 0, 0, and 1 denticle, respectively; basal denticle of the tarsungulum is subtriangular. Tarsungulum *~* 2.5× as long as wide. Basal denticle of tarsungulum with distal margin distinctly convex and *~* 0.3× as long as the basal breadth of the tarsungulum. Calyx of venom gland short, *~* 1.2× as long as wide, situated in femur.

***Leg-bearing segments*** (Fig. 7H, I). Tergites of leg-bearing segments with two paramedian sulci, becoming faint or obsolete on posterior segments. Metasternites subrectangular; without a mid-longitudinal sulcus. Ventral pores begin on leg-bearing segment 1 as a transverse group (*~* 9 pores) close to posterior margin plus two paired pore-fields (*~* 10+7 pores) close to anterior margin; on segment 4 the transverse group replaced by two paired pore-fields (*~* 16+11 pores), with two paired pore-fields (*~* 27+33 pores) close to anterior and middle regions; absent from segment 17 and posterior. Legs 1 smaller than the others; pretarsus claw-like, reaching *~* 1/4 of the length of the tarsus.

***Ultimate leg-bearing segment*** (Fig. 7J, K). Pretergite *~* 3.6× as wide as long on exposed part. Metatergite *~* 1.1× as wide as long, subtrapezoid. Metasternite *~* 1.1× as wide as long; posterior margin *~* 0.4× as wide as anterior margin; with dense setae. Coxopleuron with 19–22 pores, opening independently, scattered on the ventral and dorsal sides of coxopleuron; setae on the coxopleuron distinctly denser close to the ventral posterior edge. Ultimate leg with dense setae on ventral and lateral sides. Ultimate pretarsus claw-like; *~* 0.3× as long as tarsus. Ultimate legs swollen, with scattered setae; dense short setae on ventral sides.

***Postpedal segments*** (Fig. 7J). Intermediate sternite distinct and exposed; first genital sternite separated from pleurites by distinct sutures; gonopods biarticulate, with setae; penis conical; anal pores indistinct.

**Differences in paratype (adult female)**. Body 32–39 mm long; with 39–49 leg-bearing segments.

***Ultimate leg-bearing segment*** (Fig. 7L, M). Coxopleuron with 21–25 pores, opening independently, scattered on the ventral and dorsal sides of coxopleuron. Ultimate pretarsus claw-like; *~* 0.2× as long as tarsus. Ultimate legs slender, with scattered setae; a few setae on ventral sides.

***Postpedal segments*** (Fig. 7L). Intermediate sternite distinct and exposed; first genital pleurosternite *~* 1.8× as wide as long, posterior margin slightly concave, uniformly with sparse setae; gonopods lamina distinctly bilobate, with sparse setae; anal pores present.

##### Differences in paratype (adult male).

Body 28–35 mm long; with 45–49 leg-bearing segments.

##### Etymology.

The name refers to its type locality Lingshou county. We suggest the Chinese common name as “灵寿地蜈蚣”.

##### Remarks.

This species is also provisionally placed in the genus *Pachymerium*. Like *Pachymerium multiseriale* sp. nov., it resembles *P. ferrugineum*, but differs in possessing a narrow, subtriangular labrum and a clypeus with two indistinct, internally areolate clypeal areas, lacking the poorly defined, pigmented, weakly areolate areas near the posterior margin. It is distinguished from *P. multiseriale* by the absence of rows of dentate projections on the lateral parts of the labrum.

##### Distribution.

China (Hebei Province).

### A key to species of the genus Pachymerium*

**Table:** 

1	Coxosternite of forcipular segment with anterior denticles	**2**
–	Coxosternite of forcipular segment without anterior denticles	**22**
2	Forcipular tarsungulum with basal denticle	**3**
–	Forcipular tarsungulum without basal denticle	**20**
3	First maxillae without lappets	**19**
–	First maxillae with lappets	**4**
4	First maxillae with coxosternal lappets and telopodital lappets	**5**
–	First maxillae with telopodital lappets only (Matic et al. 1977: fig. 10c)	***P. rioindianum* Matic, Negrea & Fundora Martinez, 1977**
5	Forcipular trochanteroprefemur with one or two denticles	**6**
–	Forcipular trochanteroprefemur without denticles	***P. tyrrhenum* Verhoeff, 1934**
6	Forcipular trochanteroprefemur with two denticles	**7**
–	Forcipular trochanteroprefemur with one denticle	**10**
7	Forcipular tibia and femur with denticle	***P. armatum* Silvestri, 1905**
–	Forcipular tibia and femur without denticle	**8**
8	Coxosternite of forcipular segment with chitin lines	**9**
–	Coxosternite of forcipular segment without chitin lines	***P. capense* Attems, 1947**
9	Average number of leg pairs > 71	***P. grandiceps* (von Porat, 1893)**
–	Average number of leg pairs < 71	***P. tridentatum* Lawrence, 1960**
10	Ultimate leg with a terminal claw	**13**
–	Ultimate leg without a terminal claw	**11**
11	Average number of leg pairs > 60	**12**
–	Average number of leg pairs < 60	***P. stolli* (Pocock, 1896)**
12	Cephalic plate with transverse suture	***P. salvini* (Pocock, 1896)**
–	Cephalic plate without transverse suture	***P. cubanum* Matic, Negrea & Fundora Martinez, 1977**
13	Average number of leg pairs > 65	***P. vosseleri* Verhoeff, 1902**
–	Average number of leg pairs < 65	**14**
14	Clypeus with two paired clypeal areas	**15**
–	Clypeus without clypeal areas	**17**
15	Coxosternite of forcipular segment with chitin lines	**16**
–	Coxosternite of forcipular segment without chitin lines	***P. atticum* Verhoeff, 1901**
16	The telopodite of the first maxillae is much longer than the coxal projection (Fig. 3B)	***P. ferrugineum* (C.L. Koch, 1835)**
–	The telopodite of the first maxillae is almost as long as the coxal projection (Fig. 4F)	***P. dimorphum* sp. nov**.
17	Metasternites of leg-bearing segments with two paired pore-fields developed near the anterior margin (Fig. 7H)	***P. lingshouense* sp. nov**.
–	Metasternites of leg-bearing segments without two paired pore-fields developed near the anterior margin	**18**
18	The lateral parts of labrum with dentate projections (Fig. 6D)	***P. multiseriale* sp. nov**.
–	The lateral parts of labrum without dentate projections	***P. pereirai* Shear & Peck, 1992**
19	Coxal pores scattered on most part of the ventral side of the coxopleuron	***P. zelandicum* Attems, 1947**
–	Coxal pores close to the the margin of the metasternite (Attems 1934: fig. 103)	***P. imbricatum* Attems, 1934**
20	Metasternites of leg-bearing segments with pore-fields	**21**
–	Metasternites of leg-bearing segments without pore-fields (Dobroruka 1976: fig. 2C, D)	***P. hanzaki* Dobroruka, 1976**
21	Average number of leg pairs > 51	***P. dilottiae* Dobroruka, 1976**
–	Average number of leg pairs < 51	***P. pilosum* (Meinert, 1870)**
22	Coxosternite of forcipular segment without anterior denticles, forcipular tarsungulum without denticle	**23**
–	Coxosternite of forcipular segment without anterior denticles, forcipular tarsungulum with denticle	**24**
23	The ultimate leg with small inner serrations (Verhoeff 1943: fig. 30)	***P. serratum* Verhoeff, 1943**
–	The ultimate leg without small inner serrations	***P. idium* Chamberlin, 1960**
24	Coxosternite of forcipular segment without anterior denticles, forcipular trochanteroprefemur with denticle	**25**
–	Coxosternite of forcipular segment without anterior denticles, forcipular trochanteroprefemur without denticle	***P. multipes* (Sseliwanoff, 1881**)
25	Coxosternite of forcipular segment with chitin lines	**26**
–	Coxosternite of forcipular segment without chitin lines	***P. minutum* (Sseliwanoff, 1884)**
26	Average number of leg pairs > 63	**27**
–	Average number of leg pairs < 63	***P. proximiporum* sp. nov**.
27	Coxosternite of forcipular segment longer than wide	***P. coiffaiti* Demange, 1959**
–	Coxosternite of forcipular segment wider than long	***P. monticola* Muralewicz, 1926**

*P. brevicorne (Lucas, 1849) is probably a *Mecistocephalus* Newport, 1843, a member excluded in the present study (Lucas 1849).

## Discussion

Prior to this study, two species of *Pachymerium* had been recorded in China: *P. ferrugineum* (C.L. Koch, 1835) and *P. atticum* Verhoeff, 1901. *Pachymerium ferrugineum* was previously documented in Shanxi and Taiwan provinces. Our study, however, reveals that it is nearly ubiquitous across the Palearctic region of China and also occurs in certain parts of the Oriental region. In contrast, *P. atticum* was reported only from Shanxi Province, with no precise locality details available. The description (Takakuwa and Takashima 1949) only mentions the length of the forcipular segment and the shape of the basal denticle on the tarsungulum, which is insufficient to determine whether the specimen truly represents *P. atticum* or another species. Furthermore, Zapparoli (2002) proposed that *P. atticum* is highly likely a junior synonym of *P. ferrugineum*. This taxonomic interpretation is further supported by Popovici’s (2024) re-evaluation, which suggests that earlier records of *P. atticum* by Căpuşe (1968) from Romania differ significantly from Verhoeff’s (1901) description of this species and were likely based on misidentified juveniles of *P. ferrugineum*. Given that the originally described Greek species *P. atticum* is likely valid, we regard its reported occurrence in Shanxi as questionable. Multiple field collections in Shanxi failed to recover any specimens matching the description of *P. atticum*; instead, a new species clearly distinct from both, *P. lingshouense* sp. nov., was identified.

Given the considerable morphological variation among species of the genus *Pachymerium*, it may be inconsistent to incorporate all characteristics of every historically assigned species into the genus diagnosis. For instance, *P. brevicorne* (Lucas, 1849) lacks ultimate pretarsus; *P. atticum* Verhoeff, 1901 lacks chitin lines; *P. pereirai* Shear & Peck, 1992 lacks anterior pore-fields on the metasternites; and *P. zelandicum* Attems, 1947 lacks lappets of the first maxillae, all of which diverge from the type species *P. ferrugineum*. Moreover, the delimitation of the clypeal area within the genus is ambiguous. While in *P. ferrugineum* it is sharply bounded and non-areolate internally, illustrations of *P. tridentatum* show a poorly defined clypeal area with internal areolation. Therefore, the generic diagnosis should take the characteristics of the type species *P. ferrugineum* as the core reference, while allowing reasonable variation in certain features among other valid congeners. Based on this reasoning, we assign new species to *Pachymerium* as follows. If a new species agrees with the type species in key diagnostic characters, it is directly placed in *Pachymerium*. If a new species deviates from the type species in certain characters, but such deviations are also present in other valid congeners, it is provisionally assigned to the genus. Accordingly, *P. dimorphum* sp. nov. and *P. proximiporum* sp. nov. are consistent with the type species and are therefore assigned to *Pachymerium*. As for *P. lingshouense* sp. nov. and *P. multiseriale* sp. nov., although they differ from *P. ferrugineum* (e.g., in having a narrow labrum and different clypeus features), similar character states occur in other species of the genus. Consequently, these two new species are also provisionally placed in *Pachymerium*.

Ecologically, most Chinese *Pachymerium* species exhibit limited mobility and dispersal capacity, resulting in restricted distribution ranges. A notable exception is *P. ferrugineum*, which demonstrates a remarkably wide distribution. This species occurs regularly in urban green spaces, residential areas, and other human-modified habitats, suggesting that human activity likely facilitates its dispersal. Although *Pachymerium* is not formally recognized as an invasive genus globally and its invasion biology remains unstudied, the distribution pattern of *P. ferrugineum* strongly indicates its role as a facultative “human-assisted disperser”. Potential introduction pathways include transport via gardening soil, potted plants, and commercial freight.

Notably, individuals from different zoogeographical regions show significant morphological differences, which may lend support to this dispersal hypothesis. Specimens from the Oriental region (e.g., Guangdong, Jiangxi, Sichuan, and Yunnan provinces) are smaller, with a maximum body length of ~ 30 mm, whereas those from the Palearctic region (e.g., Heilongjiang, Jilin, and Liaoning provinces; Inner Mongolia Autonomous Region; Xinjiang Uygur Autonomous Region) are larger, reaching up to 60 mm in body length. The underlying causes of this variation remain unclear. We speculate that two factors may be involved. First, populations introduced into different urban environments may have diverged in body size due to local ecological conditions, such as temperature, humidity, or resource availability. Second, the *P. ferrugineum* populations currently found in Chinese mainland might originate from multiple source populations in the native range (Europe/Western Asia) that already differed in body size prior to introduction. Testing these hypotheses will require further experimental or population genetic studies.

Our sampling spans all seasons, with collections primarily made from typical centipede habitats such as leaf litter, rock piles, and moist soil. An analysis of the collected specimens revealed a seasonal bias in sex ratio: male *P. ferrugineum* individuals were predominantly collected in March and July. These peaks likely correspond to periods of reproductive activity in this species. If this hypothesis is confirmed, *P. ferrugineum* may continue to expand into new habitats amid ongoing globalization and urbanization. Its ecological impacts, particularly in the context of soil fauna community dynamics, warrant further investigation.

**Figure 8.**
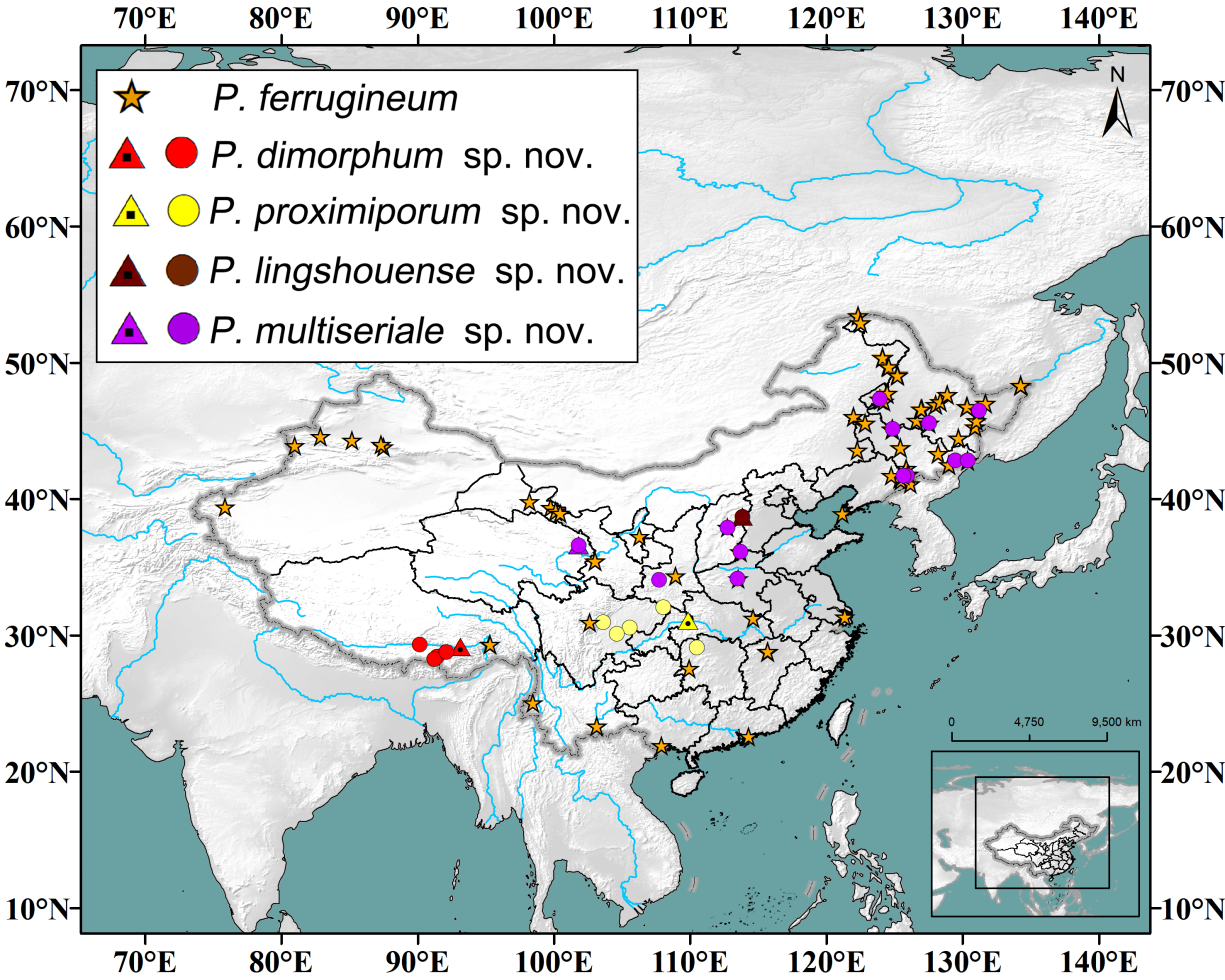
Collected localities of species of *Pachymerium* from China. Triangles represent type localities.

Finally, a biogeographic analysis shows that the northernmost, easternmost, southernmost, and westernmost records of *Pachymerium* in China are all *P. ferrugineum*, located in Heilongjiang, coastal eastern regions, Guangxi, and Xinjiang, respectively (Fig. 8). The genus exhibits remarkable elevational adaptability, ranging from near-coastal areas at seven meters (*P. multiseriale sp. nov*.) to high altitudes of 4460 meters (*P. dimorphum* sp. nov.). The broad geographic distribution of *Pachymerium* across China strongly suggests that the species diversity within this genus may be substantially underestimated, particularly along elevational gradients and in biogeographic transition zones.

## Supplementary Material

XML Treatment for Pachymerium

XML Treatment for Pachymerium ferrugineum

XML Treatment for Pachymerium dimorphum

XML Treatment for Pachymerium proximiporum

XML Treatment for Pachymerium multiseriale

XML Treatment for Pachymerium lingshouense

## Additional information

### Artificial Intelligence (AI) use

The authors accept full responsibility for the content of the manuscript, including the disclosure of any use of AI.

No AI tools were used in the preparation of this manuscript.

### Author ORCIDs

Y. Yifei https://orcid.org/0009-0008-2234-4900

Y. Xiong https://orcid.org/0009-0000-3812-9932

C. You https://orcid.org/0000-0002-3315-6090

C. Jiang https://orcid.org/0000-0003-1841-1169

### Data availability

All of the data that support the findings of this study are available in the main text or Supplementary Information.

## Supplementary materials

Supplementary material 1 List of specimens examined in this study Data type docx https://binary.pensoft.net/file/1673273This dataset is made available under the Open Database License (http://opendatacommons.org/licenses/odbl/1.0/). The Open Database License (ODbL) is a license agreement intended to allow users to freely share, modify, and use this Dataset while maintaining this same freedom for others, provided that the original source and author(s) are credited. Yifei Yu, Yuan Xiong, Chunxue You, Chao Jiang

## References

[B1] Attems CG (1903) Synopsis der Geophiliden. Zoologische Jahrbücher, Abteilung für Systematik 18: 155–302.

[B2] Attems CG (1908) Note sur les myriopodes recueillis par M. H. Gadeau de Kerville en Khroumirie et description de deux espèces et d’une varieté nouvelles provenant de cette region de la Tunisie. In: Gadeau de Kerville H (Ed.) Voyage zoologique en Khroumirie (Tunisie). Bailliere, Paris, 103–116.

[B3] Attems CG (1929) Das Tierreich. 52 Myriapoda. 1. Geophilomorpha. De Gnyter & Co., Berlin-Leipzig, 388 pp. 10.1515/9783111430638

[B4] Attems CG (1934) The Myriapoda of Natal and Zululand. Annals of the Natal Museum 7: 459–522.

[B5] Attems CG (1947) Neue Geophilomorpha des Wiener Museums. Annalen des Naturhistorischen Museums Wien 55(1944): 50–149.

[B6] Bonato L, Chagas Junior A, Edgecombe GD, Lewis JGE, Minelli A, Pereira LA, Shelley RM, Stoev P, Zapparoli M (2016) ChiloBase 2.0 – A World Catalogue of Centipedes (Chilopoda). http://chilobase.biologia.unipd.it

[B7] Bonato L, Foddai D, Minelli A, Shelley R (2004) The centipede order Geophilomorpha in the Hawaiian Islands (Chilopoda). Bishop Museum Occasional Papers 78: 13–32. http://hbs.bishopmuseum.org/fiji/pdf/bonato-etal2004.pdf

[B8] Bonato L, Edgecombe GD, Lewis JG, Minelli A, Pereira LA, Shelley RM, Zapparoli M (2010) A common terminology for the external anatomy of centipedes (Chilopoda). ZooKeys 69: 17–51. 10.3897/zookeys.69.737 PMC3088443 21594038

[B9] Bonato L, Edgecombe GD, Zapparoli M (2011) Chilopoda–Taxonomic overview. In: Minelli A (Ed.) Treatise on Zoology–The Myriapoda, vol. 1. Brill, Leiden, 363–443. 10.1163/9789004188266_020

[B10] Bonato L, Minelli A (2014) ChilopodaGeophilomorpha of Europe: a revised list of species, with taxonomic and nomenclatorial notes. Zootaxa 3770: 1–136. 10.11646/zootaxa.3770.1.1 24871280

[B11] Căpușe I (1968) Contribution à l’étude des espêces appartenant aux generes *Insigniporus* Att. et *Pachymerium* C.L. Koch (Geophilomorpha, Geophilidae). Travaux du Muséum National d’Histoire Naturelle “Gr. Antipa” 8: 699–719.

[B12] Dobroruka LJ (1976) Einige Chilopoden aus Irak. Věstník Československé společnosti zoologické 40(4): 259–262.

[B13] Dyachkov YV (2018) New data on the distribution of *Pachymerium ferrugineum* (C.L. Koch, 1835) (Chilopoda: Geophilomorpha: Geophilidae) in Central Asia. Ukrainian Journal of Ecology 8(4): 252–254.

[B14] Dyachkov YV (2022) On new records of Geophilomorpha (Chilopoda) from Middle Asia. Ecologica Montenegrina 60: 70–79. 10.37828/em.2022.60.11

[B15] Dyachkov YV (2024) An annotated checklist of the Chilopoda from Azerbaijan. Ecologica Montenegrina 71: 301–316. 10.37828/em.2024.71.33

[B16] Dyachkov YV (2025) An annotated checklist of the Chilopoda from Armenia. Ecologica Montenegrina 83: 141–151. 10.37828/em.2025.83.15

[B17] Dyachkov YV, Ali Al-Yacoub GA, Mohammed Al-Khazali AM (2023) A preliminary annotated checklist of Chilopoda from Iraq. Ecologica Montenegrina 63: 59–78. 10.37828/em.2023.63.6

[B18] Evsyukov AP, Roman Z, Dyachkov YV, Chumachenko YA, Chebotareva IP, Popov IV, Zabiyaka IY (2025) Myriapoda (Diplopoda, Chilopoda) of the Southern Cultures Park (Krasnodar Province, Southern Russia): unappreciated biodiversity. Acta Biologica Sibirica 11: 337–357. 10.5281/zenodo.15071953

[B19] Foddai D, Pereira L, Minelli A (2000) A catalogue of the geophilomorph centipedes (Chilopoda) from Central and South America including Mexico. Amazoniana 16: 59–185.

[B20] Kessler K (1874) O rysskii sorokonoshkach i shonoshkach (Scolopendridae et Geophilidae). Trudy Russkago Entomologicheskago Obshchestva 8: 28–45.

[B21] Koch CL (1835) Deutschlands Crustaceen, Myriapoden und Arachniden. In: Herrich-Schäffer GAW (Ed.) Deutschlands Insecten. Pustet, Regensburg, 136–190.

[B22] Koch CL (1847) System der Myriapoden. In: Herrich-Schäffer L (Ed.) Kritische Revision der Insectenfauna Deutschlands. Pustet, Regensburg 3, 1–270.

[B23] Lucas H (1849) Exploration scientifique de l’Algérie pendant les années 1840, 1841, 1842 publiée par ordre du Gouvernement et avec le concours d’une Commission Academique. Sciences Physiques, Zoologie. A. Bertrand, Paris, 527 pp.

[B24] Matic Z, Negrea SG, Fundora Martinez C (1977) Recherches sur les Chilopodes hypogés de Cuba. In: Résultats des Expéditions Biospéléologiques Cubano-Roumaines à Cuba. II. Editura Academiei Republicii Socialiste Romania, Bucuresti. 2: 277–301.

[B25] Minelli A, Foddai D, Pereira L, Lewis J (2000) The evolution of segmentation of centipede trunk and appendages. Journal of Zoological Systematics and Evolutionary Research 38: 103–117. 10.1046/j.1439-0469.2000.382137.x

[B26] Popovici G (2024) A review of the genus *Pachymerium* C.L. Koch, 1847 (Geophilomorpha: Geophilidae) from Romania. Zootaxa 5406(4): 588–600. 10.11646/zootaxa.5406.4.7 38480126

[B27] Takakuwa Y, Takashima H (1949) Myriapods collected in Shansi, North China. Acta Arachnologica 11: 51–69. 10.2476/asjaa.11.51

[B28] Tömösváry E (1880) Beitrag zur Kenntnis der Myriopoden Ungarns. I. Die Chilopoden.Zoologischer Anzeiger 3: 617–619.

[B29] Verhoeff KW (1901) Beiträge zur Kenntnis paläarktischer Myriopoden. XVI. Aufsatz: zur vergleichenden Morphologie, Systematik und Geographie der Chilopoden. Nova Acta Abhandlungen der Kaiserlichen Leopoldinisch-Carolinisch Deutschen Akademie der Naturforscher 77: 369–465.

[B30] Verhoeff KW (1934) Beiträge zur Systematik und Geographie der Chilopoden. Zoologische Jahrbücher. Abteilung für Systematik 66: 1–112.

[B31] Verhoeff KW (1943) Über Chilopoden der Türkei. III. Aufsatz. Zoologischer Anzeiger 143: 116–140.

[B32] Wang YM (1956) Records of myriapods on Formosa with description of new species (2). Quarterly Journal of the Taiwan Museum 9(2): 155–159.

[B33] Zapparoli M (2002) Catalogue of the centipedes from Greece. Fragmenta Entomologica 34: 1–146.

