# Supplementary material for: Four new species of Pachymerium centipedes from China (Geophilomorpha, Geophilidae)
Source: Zookeys. 2026 Jun 8;1281:265–89. doi: 10.3897/zookeys.1281.182253 (PMC13270228; doi:10.3897/zookeys.1281.182253)
Supplement: Supplementary material 1 — List of specimens examined in this study [file zookeys-1281-265_article-182253__-s001.docx]

**Supplementary file 1.** List of specimens examined in this study.

***Pachymerium ferrugineum* (C.L. Koch, 1835)**

**CHINA** **– Gansu Province** • 14♀♀ (CMMI 20230926001D–014D), Zhangye, Ganzhou Dist., Runquanhu Park (38.9544°N, 100.4718°E), 1470 m a.s.l., 26.ix.2023, leg. Tianyun Chen, Jiabo Fan & Yiying Zhao; • 1♀ (CMMI 20230927011D), Zhangye, Gaotai Dist., Yueyahu Park (39.3765°N, 99.7985°E), 1350 m a.s.l., 27.ix.2023, leg. Tianyun Chen, Jiabo Fan & Yiying Zhao; • 1♀ (CMMI 20230928002D), Jiayuguan, Guancheng (39.8031°N, 98.2256°E), 1710 m a.s.l., 28.ix.2023, leg. Tianyun Chen, Jiabo Fan & Yiying Zhao; • 1♀ (CMMI 20240921014S), Linxia Hui Autonomous Prefecture, Linxia County, near Mt. Longshoushan (35.4733°N, 103.0394°E), 2060 m a.s.l., 21.ix.2024, leg. Tianyun Chen, Jiabo Fan & Yiying Zhao; • 3♀♀ (CMMI 20230927003D–004D, -007D), Linze County, Chengnan Park (39.1379°N, 100.1525°E), 1460 m a.s.l., 27.ix.2023, leg. Tianyun Chen, Jiabo Fan & Yiying Zhao. **– Guangdong Province** • 1♂ (CMMI 20231013004), Shenzhen, Yantian Dist., Dameisha Beach (22.5885°N, 114.3006°E), 7 m a.s.l., 13.x.2023, leg. Chao Jiang. **– Guangxi Zhuang Autonomous Region** • 1♀ (CMMI 20190707020), Shangsi County, Mt. Shiwandashan (21.9044°N, 107.9038°E), 280 m a.s.l., 07.vii.2019, leg. Chao Jiang. **– Heilongjiang Province** • 1♀ (CMMI 20240822001D), Heihe, Nenjiang Town, Mergen Old Street (49.1781°N, 125.2260°E), 230 m a.s.l., 22.viii.2024, leg. Jing Zhong & Qing Li; • 1♀ (CMMI 20240819003D), Da Hinggan Range, Jiagedaqi Dist., Xingcheng Hotel (50.4123°N, 124.1394°E), 360 m a.s.l., 19.viii.2024, leg. Jing Zhong & Qing Li; • 5♀♀ (CMMI 20240820003D–007D), Da Hinggan Range, Jiagedaqi Dist., Guanghui Road (50.4122°N, 124.1351°E), 370 m a.s.l., 20.viii.2024, leg. Jing Zhong & Qing Li; • 2♀♀ (CMMI 20240820001D–002D), Da Hinggan Range, Jiagedaqi Dist., Beishan Forest Park (50.4284°N, 124.1365°E), 430 m a.s.l., 20.viii.2024, leg. Jing Zhong & Qing Li; • 6♀♀ (CMMI 20240817001D–006D), Da Hinggan Range, Mohe, Beijicun Scenic Area (53.4863°N, 122.3567°E), 280 m a.s.l., 17.viii.2024, leg. Jing Zhong & Qing Li; • 23♀♀ (CMMI 20240816004D–016D), Da Hinggan Range, Mohe, Xilinji Town, Mohe Civil Affairs Bureau (52.9758°N, 122.5294°E), 440 m a.s.l., 16.viii.2024, leg. Jing Zhong & Qing Li; • 18♀♀ (CMMI 20240817008D–024D, -027D), Da Hinggan Range, Mohe, Xilinji Town, Zhonghua Road (52.9784°N, 122.5356°E), 430 m a.s.l., 17.viii.2024, leg. Jing Zhong & Qing Li; • 15♀♀ (CMMI 20240818001D–015D), Da Hinggan Range, Mohe, Xilinji Town, Songyuan Park (52.9707°N, 122.5394°E), 450 m a.s.l., 18.viii.2024, leg. Jing Zhong & Qing Li; • 16♀♀ (CMMI 20240628012D, -014D–016D, -018D–019D, -022D, -024D–026D, -030D, -032D, -034D, -037D, -039D, -041D), Bin County, Xianglushan National Forest Park (45.5816°N, 127.5453°E), 340 m a.s.l., 28.vi.2024, leg. Yuan Xiong & Yifei Yu; • 27♀♀ (CMMI 20240629002D–028D), Harbin, Songbei Dist., Alejin Island National Wetland Park (45.8035°N, 126.6472°E), 120 m a.s.l., 29.vi.2024, leg. Yuan Xiong & Yifei Yu; • 3♀♀ (CMMI 20240822020D, -026D–027D), Nenjiang, Qianjin Town, Road Z053 (49.1051°N, 125.2510°E), 290 m a.s.l., 22.viii.2024, leg. Jing Zhong & Qing Li; • 11♀♀ (CMMI 20240720110D, -113D, -119D, -121D, -126D–131D, -133D), Jixi, Jixi Urban Forest Park (45.3082°N, 130.9323°E), 210 m a.s.l., 20.vii.2024, leg. Jiabo Fan, Jing Zhong & Feiyu Huang; • 6♀♀ (CMMI 20240715002D, -025D, -027D, -031D), Fuyuan, Fuyuan Town, Xishan site 1 (48.3658°N, 134.2856°E), 90 m a.s.l., 15.vii.2024, leg. Jiabo Fan, Jing Zhong & Feiyu Huang; • 1♀ (CMMI 20240714009D), Fuyuan, Lailailai Business Hotel (48.3644°N, 134.2903°E), 90 m a.s.l., 14.vii.2024, leg. Jiabo Fan, Jing Zhong & Feiyu Huang; • 2♀♀ (CMMI 20240715010D–011D), Fuyuan, Fuyuan Town, Fuyuan International Passenger Transport Terminal (48.3647°N, 134.2899°E), 80 m a.s.l., 15.vii.2024, leg. Jiabo Fan, Jing Zhong & Feiyu Huang; • 74♀♀ (CMMI 20240716008D–009D, -011D–015D, -018D–032D, -034D–039D, -041D–050D, -053D–055D, -057D–064D, -066D–073D, -075D, -078D–079D, -081D, -083D, -085D–087D), Fujin, Bielayinzishan Forest Park (47.0416°N, 131.7062°E), 350 m a.s.l., 16.vii.2024, leg. Jiabo Fan, Jing Zhong & Feiyu Huang; • 15♀♀ (CMMI 20240717012D–013D, -015D, -027D, -036D, -039D, -041D, -043D, 047D–049D, -053D, -068D–070D), Jiamusi, Xipu Botanical Garden (46.8046°N, 130.3108°E), 80 m a.s.l., 17.vii.2024, leg. Jiabo Fan, Jing Zhong & Feiyu Huang; • 7♀♀ (CMMI 20240717002D–005D, -007D–009D), Jiamusi, Yanjiang Park (46.8259°N, 130.3520°E), 70 m a.s.l., 17.vii.2024, leg. Jiabo Fan, Jing Zhong & Feiyu Huang; • 8♀♀ (CMMI 20240721017D–018D, -025D, -027D, -031D, -042D), Mudanjiang, Mudanfeng National Forest Park (44.4890°N, 129.7309°E), 300 m a.s.l., 21.vii.2024, leg. Jiabo Fan, Jing Zhong & Feiyu Huang; • 7♀♀ (CMMI 20240719020D, -025D, -040D–041D, -045D, -055D, -057D), Qitaihe, Xiandongshan Scenic Area (45.7821°N, 131.0186°E), 290 m a.s.l., 19.vii.2024, leg. Jiabo Fan, Jing Zhong & Feiyu Huang; • 14♀♀ (CMMI 20240823004D–009D, -011D–014D, -017D–020D), Fuyu County, Hexie Residential Area (47.7934°N, 124.4629°E), 160 m a.s.l., 23.viii.2024, leg. Jing Zhong & Qing Li; • 8♂♂47♀♀ (CMMI 20240701001D–005D, -008D–012D, -014D–025D, -027D–030D, -032D, -034D, -036D–045D, -047D–060D, -062D–064D), Qiqihar, Longsha Dist., Longsha Park (47.3496°N, 123.9454°E), 150 m a.s.l., 1.vii.2024, leg. Yuan Xiong & Yifei Yu; • 19♀♀ (CMMI 20240630001D, -003D, -005D–011D, -013D–018D, -020D–023D), Qiqihar, Zhalong National Nature Reserve (47.2026°N, 124.2468°E), 140 m a.s.l., 30.vi.2024, leg. Yuan Xiong & Yifei Yu; • 54♀♀ (CMMI 20240718009D–011D, -013D, -015D, -017D–019D, -021D, -023D–025D, -027D–031D, -034D–039D, -041D–042D, -044D–045D, -049D, -051D, -053D, -055D–056D, -059D–060D, -064D–067D, -069D–074D, -076D–077D, -079D–082D, -086D–087D, -089D, -091D), Shuangyashan, Heilongjiang Qingshan National Forest Park (46.5007°N, 131.1939°E), 260 m a.s.l., 18.vii.2024, leg. Jiabo Fan, Jing Zhong & Feiyu Huang; • 11♀♀ (CMMI 20240823001D), Fuyu County, Fuyu Forest Botanical Garden (47.7934°N, 124.4899°E), 160 m a.s.l., 23.viii.2024, leg. Jing Zhong & Qing Li; • 1♀ (CMMI 20240701093D), Qiqihar, Tiefeng Dist., Donghu Park (47.3366°N, 124.0127°E), 150 m a.s.l., 1.vii.2024, leg. Yuan Xiong & Yifei Yu; • 4♀♀ (CMMI 20240827002D), Yichun, Yimei Dist., Xing'an Forest Park (47.7074°N, 128.8970°E), 270 m a.s.l., 27.viii.2024, leg. Jing Zhong & Qing Li; • 15♀♀ (CMMI 20240825014D–016D, -019D–020D, -026D–028D, -032D–035D, -039D–041D), Suihua, Forest Botanical Garden (South Gate) (46.6159°N, 127.0397°E), 190 m a.s.l., 25.viii.2024, leg. Jing Zhong & Qing Li; • 3♀♀ (CMMI 20240825002D, -009D, -013D), Suihua, Xihu Park (46.6334°N, 126.9637°E), 170 m a.s.l., 25.viii.2024, leg. Jing Zhong & Qing Li; • 3♀♀ (CMMI 20240826007D, -013D, -024D), Tieli, Toulongshan Scenic Area (47.1734°N, 128.3666°E), 280 m a.s.l., 26.viii.2024, leg. Jing Zhong & Qing Li; • 5♀♀ (CMMI 20240826029D–031D, -033D), Tieli, Nanyuan Garden Residential Area (46.9778°N, 128.0338°E), 210 m a.s.l., 26.viii.2024, leg. Jing Zhong & Qing Li. **– Henan Province** • 1♂ (CMMI 20250325023), Yuzhou, Yingheshandi Park (34.1336°N, 113.5428°E), 100 m a.s.l., 25.iii.2025, leg. Chao Jiang & Jing Zhong; • 1♀ (CMMI 20240702025D), Linzhou, Feilong Gorge (36.1702°N, 113.6880°E), 700 m a.s.l., 02.vii.2024, leg. Jiabo Fan & Yizhan Shi. **– Hubei Province** • 1♀ (CMMI 20250329009), Hong'an County, Tumen’ao (31.2435°N, 114.6345°E), 60 m a.s.l., 29.iii.2025, leg. Chao Jiang & Jing Zhong. **– Hunan Province** • 1♂ (CMMI 20250422071), Huaihua, Zhongpo National Forest Park (27.5759°N, 109.9690°E), 280 m a.s.l., 22.iv.2025, leg. Yuan Xiong & Jing Zhong. **– Inner Mongolia Autonomous Region** • 2♀♀ (CMMI 20240821002D), Hulunbuir, Oroqen Autonomous Banner, Fanrong Road (49.7459°N, 124.6074°E), 300 m a.s.l., 21.viii.2024, leg. Jing Zhong & Qing Li; • 1♀ (CMMI 20240821001D), Hulunbuir, Oroqen Autonomous Banner, Mt. Xianglushan (49.7620°N, 124.5308°E), 300 m a.s.l., 21.viii.2024, leg. Jing Zhong & Qing Li; • 1♀ (CMMI 20250718213), Hinggan League, Horqin Right Front Banner, Horqin Park (46.0739°N, 121.9992°E), 280 m a.s.l., 18.vii.2025, leg. Jing Zhong & Chenlu Yang; • 16♀♀ (CMMI 20240704068D–070D, -072D–084D), Tongliao, Tongliao Forest Park (43.6044°N, 122.2958°E), 180 m a.s.l., 4.vii.2024, leg. Yuan Xiong & Yifei Yu. **– Jiangxi Province** • 1♂2♀♀ (CMMI 20191017002–003, 20191018022), Nanchang, Meiling National Forest Park (28.8105°N, 115.7197°E), 160 m a.s.l., 17.x.2019, 18.x.2019, leg. Chao Jiang. **– Jilin Province** • 5♀♀ (CMMI 20240702007D, -009D, -011D–012D, -016D), Baicheng, Chunhua Garden (45.6097°N, 122.8630°E), 160 m a.s.l., 02.vii.2024, leg. Yuan Xiong & Yifei Yu; • 6♀♀ (CMMI 20240704004D, -018D–019D, -059D–060D, -068D), Changchun, Jingyuetan National Forest Park (43.8017°N, 125.4582°E), 230 m a.s.l., 04.vii.2024, leg. Yuan Xiong & Yifei Yu; • 3♀♀ (CMMI 20240703062D, -064D, -067D), Songyuan, Olympic Garden (45.1284°N, 124.8539°E), 130 m a.s.l., 03.vii.2024, leg. Yuan Xiong & Yifei Yu; • 52♀♀ (CMMI 20240703001D, -003D–028D, -030D–033D, -035D–040D, -042D–047D, -049D, -051D–052D, -054D–059D), Songyuan, Ba'eda Park (45.1627°N, 124.8679°E), 120 m a.s.l., 03.vii.2024, leg. Yuan Xiong & Yifei Yu; • 3♀♀ (CMMI 20240908003D–005D), Liuhe County, Liuhe Sanxianjia National Forest Park (42.2818°N, 125.8703°E), 610 m a.s.l., 08.ix.2024, leg. Jing Zhong; • 3♀♀ (CMMI 20200914105–108), Ji'an, Yizhi Park (41.1210°N, 126.1845°E), 210 m a.s.l., 14.ix.2020, leg. Chao Jiang; • 1♀ (CMMI 20240925001D), Tonghua, Erdaojiang district, Luchanggou (41.8116°N, 126.0671°E), 840 m a.s.l., 25.ix.2024, leg. Chao Jiang; • 4♀♀ (CMMI 20240926003D), Tonghua County, Jindou Korean-Manchu Ethnic Township, Luojiagou (41.7407°N, 125.6516°E), 800 m a.s.l., 26.ix.2024, leg. Chao Jiang; • 1♀ (CMMI 20250924266), Yanbian Korean Autonomous Prefecture, Dunhua, near Gongchen Bridge (43.3770°N, 128.2263°E), 520 m a.s.l., 24.ix.2025, leg. Chao Jiang; • 1♀ (CMMI 20250923006), Yanbian Korean Autonomous Prefecture, Helong, Huahai Park (42.5502°N, 129.0042°E), 480 m a.s.l., 23.ix.2025, leg. Chao Jiang; • 8♀♀ (CMMI 20250924039, -042–043, -046, -048, -052, -054, -059), Yanbian Korean Autonomous Prefecture, Hunchun, Henandong Street (42.8657°N, 130.3756°E), 70 m a.s.l., 24.ix.2025, leg. Chao Jiang. **– Liaoning Province** • 27♀♀ (CMMI 20210905101–125), Huanren Manchu Autonomous County, Gucheng Town (41.4764°N, 125.3833°E), 390 m a.s.l., 05.ix.2021, leg. Chao Jiang; • 4♀♀ (CMMI 20230812001–002, -004, -006), Dalian, Lyushunkou Dist., Sanjianbao Seashore, (38.9360°N, 121.2043°E), 10 m a.s.l., 12.viii.2023, leg. Chao Jiang; • 1♀ (CMMI 20210907144) Xinbin Manchu Autonomous County, Yongling Town, (41.7193°N, 124.7979°E), 310 m a.s.l., 07.ix.2021, leg. Chao Jiang; • 1♂2♀♀ (CMMI 20210906117–118, -120), Huanren Manchu Autonomous County, Heigou Township, Paolugou (41.3562°N, 125.4852°E), 520 m a.s.l., 06.ix.2021, leg. Chao Jiang; • 2♀♀ (CMMI 20240707006D), Shenyang, Dongling Park (41.8360°N, 125.9960°E), 50 m a.s.l., 07.vii.2024, leg. Yuan Xiong & Yifei Yu; • 30♀♀ (CMMI 20240706001D–004D, -007D–010D, -012D, -014D–019D, -021D–024D, -026D–032D, -034D, -037D–038D, -053D), Shenyang, Shenyang National Forest Park (42.0073°N, 123.7477°E), 140 m a.s.l., 06.vii.2024, leg. Yuan Xiong & Yifei Yu. **– Ningxia Hui Autonomous Region** • 1♀ (CMMI 20230923002D), Wuzhong, Luoshan National Nature Reserve (37.2476°N, 106.3100°E), 1840 m a.s.l., 23.ix.2023, leg. Tianyun Chen, Jiabo Fan & Yiying Zhao. **– Shaanxi Province** • 1♀ (CMMI 20210331001D), Xi’an, Weiyang Dist., Xi’an North Railway Station (34.3741°N, 108.9345°E), 350 m a.s.l., 31.iii.2021, leg. Chao Jiang. **– Shanghai** • 1♀ (CMMI 20190610016), Baoshan Dist., Gucun Park (31.3498°N, 121.3708°E), 8 m a.s.l., 10.vi.2019, leg. Chao Jiang. **– Shanxi Province** • 2♀♀ (CMMI 20231030001D–002D), Taiyuan, Longcheng Forest Park (37.9285°N, 112.7630°E), 1600 m a.s.l., 30.x.2023, leg. Tianyun Chen, Yuan Xiong & Jiabo Fan. **– Sichuan Province** • 2♀♀ (CMMI 20230728009D), Aba Tibetan and Qiang Autonomous Prefecture, Xiaojin County, Dawei Town (30.9620°N, 102.6484°E), 2660 m a.s.l., 28.vii.2023, leg. Chao Jiang. **– Yunnan Province** • 1♀ (CMMI 20200605026), Tengchong, Dieshuihe Scenic Area (25.0332°N, 98.4810°E), 1600 m a.s.l., 05.vi.2020, leg. Chao Jiang; • 1♀ (CMMI 20230829001D), Gejiu, Hanting Hotel (23.3611°N, 103.1525°E), 1700 m a.s.l., 29.viii.2023, leg. Tianyun Chen & Jiabo Fan. **– Xinjiang Uygur Autonomous Region** • 2♀♀ (CMMI 20250601037–038), Kashgar Prefecture, Shufu County, G314 Highway (39.4091°N, 75.8944°E), 1290 m a.s.l., 01.vi.2025, leg. Chao Jiang; • 2♀♀ (CMMI 20250605056, -060), Urumqi, Xiaolyugu Park (43.8355°N, 87.5021°E), 800 m a.s.l., 05.vi.2025, leg. Chao Jiang; • 3♀♀ (CMMI 20250606094, -104), Changji Hui Autonomous Prefecture, Changji, Lyuzhou Subdistrict (44.0159°N, 87.3215°E), 510 m a.s.l., 06.vi.2025, leg. Chao Jiang; • 1♂ (CMMI 20250606111), Tacheng Prefecture, Shawan, Anjihai Town, G312 Highway (44.3255°N, 85.1930°E), 620 m a.s.l., 06.vi.2025, leg. Chao Jiang; • 14 ♀♀ (CMMI 20250607003, -012, -015, -017, -020, -021, -023, -026, -029, -032, -034), Bortala Mongol Autonomous Prefecture, Jinghe County, Jinghe Ecological Park (44.5991°N, 82.8784°E), 260 m a.s.l., 07.vi.2025, leg. Chao Jiang; • 2♀♀ (CMMI 20250608095), Coca Dala, Binhe Park (43.9101°N, 80.9851°E), 520 m a.s.l., 08.vi.2025, leg. Chao Jiang. **– Xizang Autonomous Region** • 1♂ (CMMI 20240309022D), Medog County, Lianhua Park (29.3253°N, 95.3317°E), 1070 m a.s.l., 09.iii.2024, leg. Chao Jiang; • 1♂ (CMMI 20240309024D), Medog County, Dexing Township (29.3270°N, 95.3033°E), 790 m a.s.l., 09.iii.2024, leg. Chao Jiang.
